# General practitioner–pharmacist collaboration to enhance deprescribing of psychotropics, sedatives, and anticholinergics among older polypharmacy patients in primary care: study protocol of a cluster-randomized controlled trial (PARTNER)

**DOI:** 10.1177/20420986251400042

**Published:** 2026-01-08

**Authors:** Annette Haerdtlein, Kerstin Bernartz, Sophie Peter, Laura K. Lepenies, Svetlana Puzhko, Yvonne Eberhardt, Michaela Maas, Stephanie Picker-Huchzermeyer, Vita Brisnik, Diana Falomir, Jochen Gensichen, Tim Steimle, Gunnar Huppertz, Michael Koller, Florian Zeman, Patrizio Vanella, Hanna M. Seidling, Achim Mortsiefer, Christiane Muth, Tobias Dreischulte

**Affiliations:** Institute of General Practice and Family Medicine, LMU University Hospital, LMU Munich, Munich, Germany; Department of General Practice and Family Medicine, Medical School OWL, Bielefeld University, Bielefeld, Germany; Institute of General Practice and Primary Care (iamag), Witten/Herdecke University, Witten, Germany; Internal Medicine IX: Department of Clinical Pharmacology and Pharmacoepidemiology/Cooperation Unit Clinical Pharmacy, Medical Faculty, University Hospital of Heidelberg, University of Heidelberg, Heidelberg, Germany; Department of General Practice and Family Medicine, Medical School OWL, Bielefeld University, Bielefeld, Germany; Center for Clinical Studies, University Hospital Regensburg, Regensburg, Germany; Institute of General Practice and Primary Care (iamag), Witten/Herdecke University, Witten, Germany; Department of General Practice and Family Medicine, Medical School OWL, Bielefeld University, Bielefeld, Germany; Institute of General Practice and Family Medicine, LMU University Hospital, LMU Munich, Munich, Germany; Institute of General Practice and Family Medicine, LMU University Hospital, LMU Munich, Munich, Germany; Institute of General Practice and Family Medicine, LMU University Hospital, LMU Munich, Munich, Germany; TK Health Insurance Fund, Hamburg, Germany; Center for Clinical Studies, University Hospital Regensburg, Regensburg, Germany; Center for Clinical Studies, University Hospital Regensburg, Regensburg, Germany; Center for Clinical Studies, University Hospital Regensburg, Regensburg, Germany; Department of Health Monitoring and Biometrics, aQua Institute, Goettingen, Germany; Internal Medicine IX: Department of Clinical Pharmacology and Pharmacoepidemiology/Cooperation Unit Clinical Pharmacy, Medical Faculty, University Hospital of Heidelberg, University of Heidelberg, Heidelberg, Germany; Institute of General Practice and Primary Care (iamag), Witten/Herdecke University, Witten, Germany; Department of General Practice and Family Medicine, Medical School OWL, Bielefeld University, Bielefeld, Germany; Institute of General Practice and Family Medicine, LMU University Hospital, LMU Munich, Nußbaumstraße 5, Munich 80336, Germany

**Keywords:** clinical trial, deprescribing, general practice, interprofessional collaboration, patient empowerment, polypharmacy, potentially inappropriate medication

## Abstract

**Background:**

Appropriate deprescribing of psychotropic, sedative, and anticholinergic potentially inappropriate medication (PSA-PIM) in older adults with polypharmacy can reduce the risk of adverse drug reactions, but is inconsistently implemented in primary care. The PARTNER intervention was designed to address challenges in PSA-PIM deprescribing at both provider and patient levels.

**Objectives:**

To evaluate the effectiveness and cost-effectiveness of the PARTNER intervention, and to understand the mechanisms of its effects.

**Design:**

Multicenter, two-arm cluster-randomized controlled trial.

**Methods and analysis:**

The study aims to recruit at least 44 clusters and 352 patients (⩾65 years old with polypharmacy (⩾5 drugs) and use of ⩾1 PSA-PIM for ⩾6 months) across three study sites in Germany. Clusters consist of one general practice and one or more community pharmacies, randomly allocated to either the PARTNER intervention or control group. The PARTNER intervention includes: (A) education for general practitioners (GPs) and pharmacists on PSA-PIM deprescribing, (B) an interprofessional workshop, (C) drug-specific empowerment brochures for patients, (D) a patient-pharmacist consultation to enhance patient empowerment, and (E) a GP-patient consultation focusing on shared decision-making. The control group receives enhanced usual care, comprising a one-off patient-pharmacist consultation for medication safety checks without a specific focus on PSA-PIM deprescribing. The intervention’s focus on PSA-PIM deprescribing is blinded to control group clusters throughout the study. The primary endpoint is a reduction in PSA-PIM exposure at 6 months (⩾0.15-point decrease in the Drug Burden Index). Secondary endpoints include falls, quality of life, healthcare utilization, and costs. The primary analysis will use a generalized linear mixed model to estimate the odds ratio for achieving the primary endpoint, adjusting for study center, age, sex, and pre-randomization PSA-PIM type and count. The process evaluation will explore the understanding of how and why the intervention succeeded or failed.

**Discussion:**

The PARTNER trial will provide evidence on the intervention’s effectiveness, efficiency, and appropriateness, informing its potential for broader implementation.

**Trial registration:**

The trial has been registered with ClinicalTrials.gov (NCT05842928) on May 6, 2023; https://clinicaltrials.gov/search?term=NCT05842928.

## Plain language summary

Improving medication safety: does collaboration between doctors and pharmacists help reduce psychotropic, sedative and anticholinergic drugs in older adults?

Older adults often take multiple medications, including psychotropic (affecting the mind), sedative (sleep-inducing), and anticholinergic (e.g. certain drugs used in the treatment of incontinence) drugs. While these drugs can be beneficial, they may also cause harmful side effects. Especially if used for a long time, the risks can outweigh the benefits. When possible, deprescribing (stopping or reducing those medications) can lower the risk of adverse effects, but can be challenging. The PARTNER study was designed to address deprescribing challenges of these target drugs by enhanced collaboration between general practitioners (GPs) and pharmacists.

The study will include at least 44 clusters, each consisting of one GP practice and one or more community pharmacies, across three German regions. The aim is to recruit at least 352 patients aged 65 years or older who are taking five or more medications, including at least one target drug for six months or longer. The clusters are randomly assigned to either the PARTNER intervention or a control group.

The PARTNER intervention includes four components: (1) education for GPs and pharmacists on deprescribing, (2) joint workshop between GPs and pharmacists, (3) drug-specific empowerment brochures for patients, (4) patient-pharmacist consultation to enhance patient empowerment, and (5) GP-patient consultation focusing on shared decision-making. In the control group, patients consult with pharmacists for general medication safety checks, without a specific focus on deprescribing target drugs.

The primary goal is to see if the PARTNER intervention can reduce patients’ exposure to target drugs after six months. Further goals include assessing intervention effects on falls, quality of life, healthcare utilization, and costs.

If effective, the PARTNER intervention could provide a practical approach to safely reducing psychotropic, sedative and anticholinergic drugs in older adults through enhanced GP-pharmacist collaboration. This could improve patient safety and health outcomes.

## Introduction

Polypharmacy, which is commonly defined as the simultaneous use of five or more drugs,^1^ is predominantly managed in primary care and substantially increases the risk of adverse drug reactions (ADRs), which may cause serious harm. For instance, a recent meta-analysis found that 8.3% of emergency hospital admissions are due to ADRs, half of which were deemed preventable.^2^

To mitigate the risks associated with polypharmacy, guidelines recommend comprehensive medication reviews to evaluate the need, effectiveness, safety, and patient-centeredness of medication regimens in the context of individualized treatment goals.^3,4^ Such medication reviews can lead to deprescribing—the process of discontinuing or reducing the dose of drugs that provide limited benefit to the patient or expose them to unacceptable risks. Deprescribing, supervised by a healthcare professional, aims to manage polypharmacy and improve outcomes.^5–7^

Over the last three decades, numerous lists of potentially inappropriate medication (PIM) have been developed. These include drugs that should be avoided in older people, either because their risks outweigh their benefits overall or in specific clinical situations, and may therefore be prioritized targets for deprescribing.^8^

However, deprescribing remains underutilized in primary care. A number of interventions to promote deprescribing (where appropriate) have been developed and evaluated with mixed results.^9^ For example, the German RIME intervention provided education on a broad range of PIMs to general practitioners (GPs) and practice teams, as well as paper-based reminder cards, but showed no effect on the use of 80 PIMs.^10^ Similarly, the Irish SPPiRE trial reported no impact of GP education on polypharmacy reviews in reducing the use of 34 PIMs.^11^ In contrast, the Scottish DQIP intervention, which focused solely on nonsteroidal anti-inflammatory drugs and antiplatelets, demonstrated significant reductions in targeted prescribing and associated clinical outcomes. This was achieved through an IT-based case-finding tool combined with education and financial incentives for prescribers.^12^ Yet, a similar intervention in Scotland targeting antipsychotics showed no impact on PIM rates.^13^ The EMPOWER trial found that provision of empowerment brochures to long-term users of benzodiazepines led to significantly higher cessation rates in the intervention versus the usual care control arm.^14^ These findings may suggest that prescriber education alone may not be sufficient, that interventions targeting a narrower range of PIMs may be more effective than those with a broader scope, and that deprescribing interventions may be effective for certain PIMs but not for others.

The PARTNER project focuses on psychotropic, sedating, and anticholinergic PIMs (PSA-PIMs). PSA-PIMs have in common that they can trigger or worsen geriatric syndromes, such as falls/fractures, cognitive impairments/delirium, and malnutrition, all of which significantly affect quality of life for older people.^8,9,15^ While deprescribing PSA-PIMs may therefore offer substantial benefits, it also poses distinct challenges.^15^ Although these drugs may provide subjective relief, some PSA-PIMs (e.g., hypnotics used to treat insomnia) are addictive.^16,17^ In addition, abrupt cessation of all PSA-PIMs may cause withdrawal symptoms, including symptom rebound (e.g., insomnia), which initially prompted their use.^18–20^ As a consequence, patients may be reluctant to agree to PSA-PIM deprescribing or to stop deprescribing attempts prematurely, while GPs may be reluctant to engage in a complex and time-consuming deprescribing process.

The PARTNER intervention is a complex intervention targeting PSA-PIMs, aiming to support deprescribing of these drugs via healthcare provider education, patient empowerment, and interprofessional collaboration. This study protocol describes the evaluation of the PARTNER intervention through (1) a cluster-randomized controlled trial (cRCT) to evaluate its effectiveness and potential undesired consequences, (2) a health economic evaluation to examine benefits in relation to costs, and (3) a process evaluation to explore why and how the intervention succeeded or failed.

## Methods

### Cluster-randomized controlled trial

The protocol follows the *Standard Protocol Items: Recommendations for Interventional Trials* (SPIRIT) 2025 statement^21^ (see Supplemental Material). The study protocol of the cRCT has been developed, and the findings will be reported in line with the *Consolidated Standards of Reporting Trials (CONSORT) statement: extension to cluster-randomized trials*, respectively.^22,23^

#### Aims and objectives

The primary objective of the cRCT is to evaluate the effectiveness of the PARTNER intervention in reducing the exposure to PSA-PIMs at the patient level at 6 months follow-up, with 1 month being defined as 30 days. Secondary objectives comprise evaluation of the intervention effect on PSA-PIM exposure at 12 months, on different groups of PSA-PIMs, on prevalent (“old”) and incident (“new”) prescribing of PSA-PIMs, on other (non-PSA) PIMs and potential prescribing omissions (PPOs), and desired and undesired patient-reported and clinical outcomes.

#### Study design

The trial uses a multicenter, cluster-randomized design with two parallel arms and a 1:1 allocation ratio. The intervention arm receives the PARTNER intervention, while the control arm undergoes enhanced treatment as usual (eTAU). To minimize patient selection bias, clusters are blinded to their treatment allocation and the intervention’s focus on PSA-PIMs until patient recruitment is finalized in each cluster. After randomization, only the intervention arm will be unblinded. The PARTNER intervention includes cluster-level components (outlined below), and a patient-randomized design was deemed unsuitable due to the high risk of contamination in the control arm, making cluster randomization the preferred approach.

#### Blinding

To avoid patient selection bias, the clusters are blinded to the target drugs (PSA-PIM) of the intervention until they are informed of their allocated treatment arm. To reduce contamination bias, control arm clusters remain unaware of the intervention’s specific focus on PSA-PIMs throughout the trial. In the intervention arm, the blinding is broken as soon as clusters are informed of their allocated group and provided access to educational material targeting PSA-PIM deprescribing.

#### Participants

The clusters (i.e. the units of randomization) are collaboration units formed for the purposes of this trial. Each cluster consists of one GP practice and one or more community pharmacies, which provide care for a patient enrolled in this trial.

##### Cluster eligibility criteria

GP practices are recruited in three study regions in Germany (Upper Bavaria with study center Munich, Western Westphalia with study center Witten, Eastern Westphalia with study center Bielefeld). Community pharmacies located in the vicinity of recruited GP practices are invited to participate in the study. To avoid contamination between the study arms, the recruited cooperation units are selected in such a way that overlap of clusters (i.e., patients being managed by more than one participating cluster) can be ruled out, for example by maintaining sufficient geographic distance between recruited clusters. Table 1 provides detailed eligibility criteria for GP practices and pharmacies.

**Table 1.** Eligibility criteria for clusters (cooperation units of one GP practice and one or more community pharmacies).

Inclusion criteria	Exclusion criteria
A. GP practices	
• Provision of medical care to patients under statutory (public) health insurance • Practice size/structure, which is expected to have a sufficient number of eligible patients	• Specialist practice for unconventional medical treatments • Specialist practice for special indications (e.g., HIV)
B. Community pharmacies	
• —	• No suitable premises for conducting confidential patient consultations

GP, general practitioner.

##### Patient eligibility criteria

Eligible patients are recruited by GP practices, who are instructed to recruit between 6 and 15 patients (to confine heterogeneity of cluster sizes). The target population comprises older people with polypharmacy receiving outpatient care from GP practices and community pharmacies, who take at least one PSA-PIM, and who are mobile and able to provide information for the study via telephone. Table 2 lists the detailed eligibility criteria.

**Table 2.** Eligibility criteria for patients.

Inclusion criteria	Exclusion criteria
• Age ⩾65 years • Patient is capable of giving consent • GP contact in the quarter prior to inclusion • Current use of ⩾5 active substances, including ⩾1 PSA-PIM* with a treatment duration of ⩾6 months • Willingness to select a regular pharmacy for the duration of the study • Consent to data exchange between GP and community pharmacy	• Terminal illness (life expectancy <6 months) • Current treatment of pain associated with cancer • Other serious physical illness or mental distress (e.g., bereavement) that makes participation in the study impossible (according to the GP’s assessment) • Psychiatric illness or addiction that makes participation in the study impossible (according to the GP’s assessment) • Unable to meet the requirements of the study (participation in telephone or written questionnaires, visits to the GP practice or community pharmacy, alone or with the help of caregivers for physical infirmity) • Current participation in research projects on medication safety or geriatric medicine

* Hypnotics (benzodiazepines, z-drugs), opioids, gabapentinoids, antipsychotics, antidepressants, anticholinergic urospasmolytics. Where PSA-PIMs are prescribed as required, the inclusion criterion is met if the patient takes the drug at least twice per week.

GP, general practitioner; PSA-PIM, psychotropic, sedating, and anticholinergic potentially inappropriate medication.

##### Cluster recruitment

During recruitment, clusters are provided with the following information: “The PARTNER study aims to enhance medication safety for elderly patients with multiple medications and potential medication risks by fostering closer collaboration between GP practices and one or more partnering pharmacies. In both the intervention (IG) and control groups (CG), patients will receive an evaluation of their current medication and a risk assessment during a planned visit to the pharmacy. In the intervention group, there will also be an interprofessional workshop involving both the GP practices and pharmacies, as well as an additional patient visit to the GP practice.” This communication is designed to generate interest while maintaining blinding by not disclosing the intervention’s specific focus on PSA-PIM deprescribing.

##### Patient screening and recruitment

GP practices will generate screening lists of patients meeting all the following inclusion criteria: age ⩾65 years, at least one GP visit in the quarter prior to inclusion, and use of ⩾5 active substances. The patient’s current medication will then be entered into a software tool, which applies an algorithm to identify the use of ⩾1 PSA-PIM. The software only notifies the GP practice whether the patient meets the inclusion criteria, without specifying the qualifying medication. To minimize selection bias, the remaining eligible patients are randomly assigned numbers and listed in ascending order. Starting with the first patient on the list, the GP verifies the exclusion criteria. Patients who remain eligible are then invited by the GP practice to participate in the study. If interested, informed consent will be obtained by the GP.

#### Interventions

**Figure 1.**
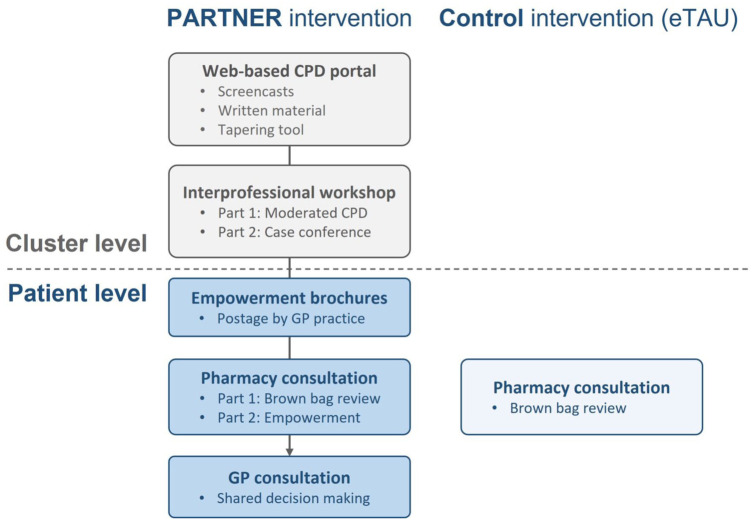
Intervention components in the two arms of the PARTNER trial. CPD, continuing professional development; eTAU, enhanced treatment as usual; GP, general practitioner.

The cRCT compares the PARTNER intervention with an intervention that is consistent with eTAU. Figure 1 shows the intervention components in the PARTNER intervention and eTAU control arms.

##### Rationale and components of the PARTNER intervention arm

The PARTNER intervention has been designed to address key barriers to deprescribing PSA-PIMs. One such barrier is inertia, that is, in this context, a reluctance to deprescribe PSA-PIMs without an acute reason, such as ADRs.^24,25^ At the level of healthcare professionals, this is exacerbated by competing priorities, time pressure, and a lack of knowledge on how best to deprescribe (e.g., the design and practical implementation of tapering schemes) as well as patient resistance.^26,27^ The latter can be fueled by patient misconceptions about the benefits and risks of PSA-PIMs.^28^ To overcome these barriers, the PARTNER intervention includes components aiming to educate and motivate healthcare professionals (via a web-based continuing professional development (CPD) portal and a moderated live CPD session) as well as patients (via empowerment brochures). In addition, there are components aiming to facilitate its practical implementation via tools, interprofessional case conferences, and delegation of patient empowerment to community pharmacies.

GPs and pharmacists volunteer to participate and to implement either the PARTNER intervention or the control intervention. While no financial incentives are provided as components of the intervention itself, GPs and pharmacists are compensated for their time and effort. Specifically, GP practices and community pharmacies in the intervention group receive €180 per patient enrolled in their cluster, while those in the control group receive €120 per patient. The higher compensation in the intervention group reflects the additional time, documentation requirements, and complexity associated with the intervention. Participating providers in the intervention group are required to complete a short questionnaire for each intervention component, enabling monitoring of compliance with the study protocol. However, it is important to note that the study protocol does not compel participants in either arm to undertake a deprescribing attempt.

Table 3 maps the intervention components to specific functions and elements of the COM-B model.^29^ According to COM-B, behavior (e.g., of healthcare professionals or patients) is a result of the interaction between three essential components: Capability (C), Opportunity (O), and Motivation (M), which together determine whether a Behavior (B) will occur. This categorization facilitates a higher-level understanding of which interventions are suitable and effective for addressing particular healthcare challenges.

**Table 3.** Mapping components of the PARTNER intervention to the COM-B model.^29^.

COM-B component	Intervention element (target group)	Intervention function and rationale
Psychological capability (C-Ps)	- Web-based CPD portal/screencasts (HCP) - Interprofessional workshops (HCP) - Training in shared decision-making (HCP)	*Education*: Improves healthcare professionals’ knowledge and skills in deprescribing, enhancing their psychological capability to make informed decisions
Social opportunity (O-So)	- Interprofessional workshops (HCP) - Collaboration between GPs and community pharmacies (HCP)	*Environmental restructuring*: Creates opportunities for healthcare professionals to collaborate and share knowledge, fostering a supportive environment for deprescribing
Physical opportunity (O-Ph)	- Web-based tapering support tool (HCP) - Pharmacy consultations (HCP)	*Environmental restructuring*: Provides healthcare professionals with tools and resources to implement deprescribing practically, such as tapering support tools and consultation frameworks
Reflective motivation (M-Re)	- Motivational screencasts (HCP) - Empowerment brochures (Pat) - Shared decision-making processes in GP consultations (HCP/Pat)	*Education*: Motivates both healthcare professionals and patients by increasing awareness about the benefits and risks of deprescribing, prompting reflective decision-making
Automatic motivation (M-Au)	- Empowerment brochures emphasizing gradual tapering benefits (Pat) - Positive emotional framing in pharmacy consultations (Pat)	*Incentivization*: Engages patients’ automatic motivation by focusing on emotional benefits and reinforcing positive associations with deprescribing, making the process feel less intimidating

COM-B, Capability, Opportunity, Motivation—Behavior; HCP, healthcare professionals; Pat, patients.

Web-based CPD portal (cluster level).

Clusters randomized to the intervention arm receive access to a web-based portal with the following resources on PSA-PIM deprescribing:

- Screencasts aiming to overcome inertia and increase motivation for deprescribing. This is achieved by emphasizing the importance of a primary prevention approach to avoid PSA-PIM-associated harm and by explaining how the PARTNER intervention provides resources to facilitate it. Five short screencasts (between 5 and 12 min) are provided, in which an academic GP and an academic pharmacist provide information and guidance on (i) the rationale of the intervention’s focus on PSA-PIMs, (ii) intervention components and procedures, (iii) PSA-PIM tapering schemes, (iv) tools for communication with patients (including teach back and shared decision-making), (v) tools for conducting medication reviews.
- Downloadable/printable educational material (brief and detailed versions) on PSA-PIM benefits and risks, when to deprescribe them and when not to, as well as PSA-PIM withdrawal symptoms and how to manage them.
- Link to a (i) web-based tapering support tool that offers general information about tapering PSA-PIM and synthesized information on current guidelines and recommendations, and calculates possible tapering schemes and (ii) the accompanying PSA-PIM list, which provides an overview of the oral dosage forms, dosage strengths, and divisibility of pharmaceutical products available in Germany and is intended to support the practical implementation of the planned tapering schemes. Downloadable/printable guidance to support community pharmacists in conducting medication reviews, including a PIM/PPO checklist.

Interprofessional workshop (cluster level).

The interprofessional workshop aims to deepen, focus, and strategically plan the collaboration between GP practices and community pharmacies of each cluster randomized to the intervention arm. In preparation for the workshop, clusters are asked to familiarize themselves with the resources provided in the web-based portal (see above), and GPs are asked to provide community pharmacists with relevant clinical information on included patients using standardized templates (medication, diagnoses, selected laboratory results). The workshop itself has a planned duration of up to 2 h and consists of two parts. Part 1 (intervention training) is moderated by the study team. The session aims to ensure that GP practices and community pharmacies understand the rationale behind the intervention’s focus on PSA-PIMs. It facilitates introductions between team members, clarifies their respective roles and responsibilities, and familiarizes participants with the resources and tools available on the web-based portal. This component is designed to be flexible, allowing the study team to adapt the content and emphasize topics of particular relevance to each cluster. Part 2 (case conference), which is conducted independently of the study team, enables GPs and community pharmacists to discuss each study patient. Together, they decide whether deprescribing should be attempted and identify specific drugs to target for deprescribing. This structured yet adaptable approach ensures that each cluster is equipped to collaborate effectively while tailoring the intervention to meet their unique needs.

Empowerment brochures (patient level).

In the PARTNER intervention arm, the GP practice sends drug-specific and layperson-friendly empowerment brochures to patients selected in the interprofessional workshop (see above), unless the respective GP and pharmacist concluded that PSA-PIM deprescribing should not be attempted for a specific patient. To encourage patients to engage with the brochure, each patient also receives a personalized letter from their GP practice asking them to read the brochure at home and bring it along to their pharmacy visit. Seven different brochures with a focus on the following drugs and indications are available:

- benzodiazepines and z-drugs
- antipsychotics
- antidepressants in the treatment of sleeping problems
- antidepressants in the treatment of depression
- opioids
- gabapentinoids in the treatment of chronic pain
- anticholinergic antispasmodics in the treatment of incontinence

The content of the empowerment brochures is based on instruments that were successfully used in a Canadian cluster-randomized trial (EMPOWER) on the deprescribing of benzodiazepines^14^ and is designed to deliver the following educational messages: (a) patients realistically assess the benefits and risks of PSA-PIM use; (b) patients understand that adverse events following any previous attempts to discontinue PSA-PIM are likely an expression of habituation and are usually temporary in nature; (c) patients understand that there may be effective and sustainable nonpharmacological alternatives for the treatment of symptoms treated with PSA-PIM; (d) patients understand that discontinuing PSA-PIMs gradually can minimize the risk of any adverse withdrawal effects. Information on the extent to which patients engaged with the empowerment brochures and understood their content will be documented in two ways: first, through the pharmacist’s assessment after the patient’s pharmacy visit, including the empowerment conversation; and second, through a patient survey conducted at the end of the study as part of the process evaluation (see below).

Pharmacy consultation (patient level).

All patients are invited to attend a consultation with the designated community pharmacist within the premises of the pharmacy and to bring along all medicines they are currently taking, as well as any empowerment brochures they have received. The consultation is planned to last approximately 1 h and consists of two parts. In part 1 (brown bag review), the community pharmacist checks the current medication for medication-related problems (MRPs) on the basis of the information provided by the GP practices and a structured patient discussion. To support MRP identification, the pharmacist is encouraged to use the PIM/PPO checklist provided in the web-based portal (see above). The pharmacist’s findings of the medication review are documented on standardized templates and sent to the GP practice. In part 2 (empowerment), the community pharmacist draws attention to any empowerment brochures sent to the patient and aims to ensure that their key educational messages are understood by the patient.

GP consultation (patient level).

GP and patient agree on any medication changes and how to implement them (including a tapering and follow-up plan). To increase the chance of successful and sustained PSA-PIM deprescribing, GPs are encouraged (via web-based portal and moderated interprofessional workshop) to apply shared decision-making and to reiterate and emphasize to their patients that withdrawal symptoms are usually mild and temporary, and that tapering schemes can be adapted to minimize any experienced adverse effects.

##### Rationale and components of the eTAU control arm

The study design and the control intervention were chosen to evaluate the additional benefit of an in-depth, patient-centered collaboration between GPs and pharmacists, focusing on PSA-PIM (PARTNER intervention), compared to a generic approach not focused on particular target drugs and without structured pharmacist-GP collaboration. The control arm was designed to align with enhanced routine practice in Germany, where community pharmacists may be reimbursed for conducting “medication reviews for patients with polymedication” (with or without involving GPs at their discretion) since June 2022,^30^ although implementation remains patchy.^31^

Pharmacy consultation (patient level).

The only component of the eTAU control arm is a brown bag review (as in the intervention arm). Community pharmacists are instructed to provide a “medication check-up” with any follow-up at their discretion. In contrast to the intervention arm, the study team neither provides guidance nor tools to support these reviews, nor is there a requirement to feed back any findings to GP practices.

Table 4 provides the timeline for intervention components and data collection for each cluster.

**Table 4.** Timeline for intervention components and data collection for each cluster.

Time	Activity	Parties involved in activity			
		Research team	GP	Pharmacy	Patients
Minus 5 months	Recruitment of GP practice and collaborating community pharmacy	✓			
Minus 3 months	Study training		✓	✓	
Minus 3 months	Patient screening		✓		
Minus 2 months	Patient recruitment		✓		
Minus 1 month	Randomization	✓			
Minus 1 month	Data collection at T_0_: Medication, Health-Related Resource Use^a^, HRQoL^b^, Insomnia^c^, Cognition^d^, Adverse Drug Reactions^e^	✓			✓
Minus 1 month	Provide access to educational portal*	✓			
Minus 1 month	Familiarization with educational portal content*		✓	✓	
Minus 2 weeks	Interprofessional workshop*	✓	✓	✓	
Minus 2 weeks	Postage of empowerment brochures*		✓		
Reference	Pharmacy consultation			✓	✓
Plus 2 weeks	GP consultation*		✓	✓	
Plus 3 months	Data collection at T_1_: Health-Related Resource Use^a^, HRQoL^b^	✓			✓
Plus 6 months	Data collection at T_2_: Medication, Health-Related Resource Use^a^, HRQoL^b^, Insomnia^c^, Cognition^d^, Adverse Drug Reactions^e^	✓			✓
Plus 9 months	Data collection at T_3_: Health-Related Resource Use^a^, HRQoL^b^	✓			✓
Plus 12 months	Data collection at T_4_: Medication, Health-Related Resource Use^a^, HRQoL^b^, Insomnia^c^, Cognition^d^, Adverse Drug Reactions^e^	✓			✓

*Measurement tools*: ^a^Questionnaire for Health-Related Resource Use in an Elderly Population (FIMA), ^b^EuroQol 5-Dimension 5-Level (EQ-5D-5L) questionnaire, ^c^Regensburg Insomnia Scale (RIS), ^d^Verbal Fluency Test, ^e^Patient-reported ADR questionnaire.

* Intervention arm only.

GP, general practitioner; HRQoL, Health-Related Quality of Life.

#### Outcomes

##### Data collection

Participating GP practices, pharmacies, and patients serve as data sources. GP practices document relevant data to characterize the patient collective upon inclusion in the study, and GP practices and pharmacies document all study-related patient contacts during the follow-up in the intervention group. All study endpoints listed in Tables 4 to 9 are collected by trained study personnel via patient interviews. The follow-up period is 12 months, with data collection taking place at five points in time:

- Data collection for T_0_ is at randomization.
- T_1_, T_2_, T_3_, and T_4_ are defined as 3, 6, 9, and 12 months after the pharmacy consultation in each arm, respectively.
- In case the pharmacy consultation does not take place within 3 months after randomization (protocol deviation), T_1_, T_2_, T_3_, and T_4_ are defined as 6, 9, 12, and 15 months after randomization.

**Table 5.** Primary outcome—PSA-PIM deprescribing.

No.	Source population	Parameter	Time of measurement*
Impact on DBI response			
Primary outcome	All study participants	Number of patients in source population with a reduction in the DBI (including all PSA-PIMs) of ⩾0.15	T_2_ vs T_0_

The outcome is the rate of parameter/source population in the intervention versus the control arm.

* Data collection for T_0_ is at randomization. T_1_, T_2_, T_3_, and T_4_ are defined as 3, 6, 9, and 12 months after the pharmacy consultation in each arm, respectively. In case the pharmacy consultation does not take place within 3 months after randomization (protocol deviation), T_1_, T_2_, T_3_, and T_4_ are defined as 6, 9, 12, and 15 months after randomization.

DBI, Drug Burden Index; PSA-PIM, psychotropic, sedating, and anticholinergic potentially inappropriate medication.

**Table 6.** Secondary outcomes (SO)—PSA-PIM deprescribing and use.

No.	Source population	Parameter	Time of measurement*
Sustained and stratified impact on DBI response			
SO1	All study participants	Number of patients in source population with a reduction in the DBI (including all PSA-PIMs) of ⩾0.15	• T_4_ vs T_0_
SO2	Study participants with chronic pain psychotropics at baseline	Number of patients in source population with a reduction in the DBI (including chronic pain psychotropics only) of ⩾0.15	• T_2_ vs T_0_ • T_4_ vs T_0_
SO3	Study participants with mental health psychotropics at baseline	Number of patients in source population with a reduction in the DBI (including mental health psychotropics only) of ⩾0.15	• T_2_ vs T_0_ • T_4_ vs T_0_
SO4	Study participants with sedative-hypnotics at baseline	Number of patients in source population with a reduction in the DBI (including sedative-hypnotics only) of ⩾0.15	• T_2_ vs T_0_ • T_4_ vs T_0_
SO5	Study participants with anticholinergic urological antispasmodics at baseline	Number of patients in source population with a reduction in the DBI (including anticholinergic urological antispasmodics only) of ⩾0.15	• T_2_ vs T_0_ • T_4_ vs T_0_
Impact on overall number of PSA-PIMs			
SO6	All study participants	Number of PSA-PIMs taken by patients in source population	• T_2_ vs T_0_ • T_4_ vs T_0_
Impact on baseline PSA-PIM use			
SO7	All study participants	Patients in source population with reduced number of “old” (i.e., present at baseline) PSA-PIMs	• T_2_ vs T_0_ • T_4_ vs T_0_
Impact on new PSA-PIM prescribing			
SO8	All study participants	Patients in source population with “new” (i.e., different from baseline) PSA-PIMs	• T_2_ vs T_0_ • T_4_ vs T_0_

The outcome is the rate of parameter/source population in the intervention versus the control arm, unless specified otherwise

* Data collection for T_0_ is at randomization. T_1_, T_2_, T_3_, and T_4_ are defined as 3, 6, 9, and 12 months after the pharmacy consultation in each arm, respectively. In case the pharmacy consultation does not take place within 3 months after randomization (protocol deviation), T_1_, T_2_, T_3_, and T_4_ are defined as 6, 9, 12, and 15 months after randomization.

DBI, Drug Burden Index; PSA-PIM, psychotropic, sedating, and anticholinergic potentially inappropriate medication; SO, secondary outcomes.

**Table 7.** Secondary outcomes—other PIMs and PPOs.

No.	Source population	Parameter	Time of measurement*
SO9	All study participants with other PIMs at baseline	Number of resolved PIMs in source population	• T_2_ vs T_0_ • T_4_ vs T_0_
SO10	All study participants with PIMs increasing bleeding risk at baseline	Number of resolved PIMs increasing bleeding risk in source population	• T_2_ vs T_0_ • T_4_ vs T_0_
SO11	All study participants with PIMs increasing risk of acute kidney injury at baseline	Number of resolved PIMs increasing risk of acute kidney injury in source population	• T_2_ vs T_0_ • T_4_ vs T_0_
SO12	All study participants with PIMs increasing risk of cardiac decompensation and/or bradycardia at baseline	Number of resolved PIMs increasing risk of cardiac decompensation and/or bradycardia in source population	• T_2_ vs T_0_ • T_4_ vs T_0_
SO13	All study participants with PIMs increasing risk of falls, fractures, delirium at baseline	Number of resolved PIMs increasing risk of falls, fractures, delirium in source population	• T_2_ vs T_0_ • T_4_ vs T_0_
SO14	All study participants with PIMs increasing risk of hypoglycemia at baseline	Number of resolved PIMs increasing risk of hypoglycemia in source population	• T_2_ vs T_0_ • T_4_ vs T_0_
SO15	All study participants with drug-drug interactions at baseline	Number of resolved drug-drug interactions in source population	• T_2_ vs T_0_ • T_4_ vs T_0_
SO16	All study participants with drug-risk factor interactions at baseline	Number of resolved drug-risk factor interactions in source population	• T_2_ vs T_0_ • T_4_ vs T_0_
SO17	All study participants with potential prescribing omissions at baseline	Number of resolved potential prescribing omissions in source population	• T_2_ vs T_0_ • T_4_ vs T_0_

The outcome is the rate of parameter/source population in the intervention versus control arm

* Data collection for T_0_ is at randomization. T_1_, T_2_, T_3_, and T_4_ are defined as 3, 6, 9, and 12 months after the pharmacy consultation in each arm, respectively. In case the pharmacy consultation does not take place within 3 months after randomization (protocol deviation), T_1_, T_2_, T_3_, and T_4_ are defined as 6, 9, 12, and 15 months after randomization.

DBI, Drug Burden Index; PIM, potentially inappropriate medication; PPO, potential prescribing omission; SO, secondary outcomes.

**Table 8.** Secondary outcomes—patient-reported outcomes.

No.	Source population	Parameter	Time of measurement*
SO18	All study participants	Cognition (Verbal Fluency Test) in source population	• T_0_, T_2_, T_4_
SO19	All study participants	Insomnia (Regensburg Insomnia Scale) in source population	• T_0_, T_2_, T_4_
SO20	All study participants	Quality of life (EQ-5D-5L) in source population	• T_0_, T_1_, T_2_, T_3_, T_4_

* Data collection for T_0_ is at randomization. T_1_, T_2_, T_3_, and T_4_ are defined as 3, 6, 9, and 12 months after the pharmacy consultation in each arm, respectively. In case the pharmacy consultation does not take place within 3 months after randomization (protocol deviation), T_1_, T_2_, T_3_, and T_4_ are defined as 6, 9, 12, and 15 months after randomization.

EQ-5D-5L, EuroQol 5-Dimension 5-Level questionnaire; SO, secondary outcomes.

**Table 9.** Secondary outcomes—clinical outcomes.

No.	Source population	Parameter	Time of measurement*
SO21	All study participants	Number of falls (reported in fall diaries) in source population	• T_1_, T_2_, T_3_, T_4_
SO22	All study participants	Number of emergency hospital admissions due to falls in source population	• T_1_, T_2_, T_3_, T_4_
SO23	All study participants	Number of adverse drug reactions in source population	• T_0_, T_2_, T_4_

* Data collection for T_0_ is at randomization. T_1_, T_2_, T_3_, and T_4_ are defined as 3, 6, 9, and 12 months after the pharmacy consultation in each arm, respectively. In case the pharmacy consultation does not take place within 3 months after randomization (protocol deviation), T_1_, T_2_, T_3_, and T_4_ are defined as 6, 9, 12, and 15 months after randomization.

SO, secondary outcomes.

Hospitalizations, use of medical services, and quality of life estimates are collected at all five time points. Falls/fall injuries, hospital admissions (with outpatient or inpatient care), and contacts with GPs or specialist doctors are also documented in patient diaries to minimize recall bias. The use of a patient diary is regularly checked by study personnel. All other endpoints are recorded at T_0_, T_2_, and T_4_ (see Tables 5–10). Participant retention and complete follow-up are promoted by the regular phone calls (in intervals of 3 months) to collect primary and secondary outcome data. In case of study discontinuation, the stated reason(s) will be documented.

**Table 10.** Health economic outcomes.

No.	Benefit	Cost	Time of measurement*
HEO1	Primary Outcome (reduction in Drug Burden Index of ⩾0.15)	Questionnaire for Health-Related Resource Use in an Elderly Population (FIMA):	T_0_, T_2_, T_4_
HEO2	Quality of life (EQ-5D-5L Score)	- Outpatient medical care - Inpatient medical care - Drug costs	T_0_, T_1_, T_2_, T_3_, T_4_
HEO3	Quality of life (EQ-VAS)	- Provision of remedies and aids - Outpatient care/rehabilitation	T_0_, T_1_, T_2_, T_3_, T_4_
HEO4	Falls (Patient Diary)	- Resources GP practice/pharmacy (personnel, infrastructure, and material)	T_1_, T_2_, T_3_, T_4_
HEO5	Cognition (Verbal Fluency Test Score)		T_0_, T_2_, T_4_

* Data collection for T_0_ is at randomization. T_1_, T_2_, T_3_, and T_4_ are defined as 3, 6, 9, and 12 months after the pharmacy consultation in each arm, respectively. In case the pharmacy consultation does not take place within 3 months after randomization (protocol deviation), T_1_, T_2_, T_3_, and T_4_ are defined as 6, 9, 12, and 15 months after randomization.

EQ-5D-5L, EuroQol 5-Dimension 5-Level questionnaire; EQ-VAS, EuroQol Visual Analogue Scale; HEO, health economic outcomes.

##### Primary outcome

The primary endpoint of the cluster-randomized study is a reduction in PSA-PIM exposure at the patient level after 6 months of follow-up, which is measured using the Drug Burden Index (DBI) approach.^32^ The primary endpoint measures the responder rate in the intervention versus control arm, where response is defined as a reduction in the DBI by ⩾0.15 points (see Table 5).

**Figure 2.**
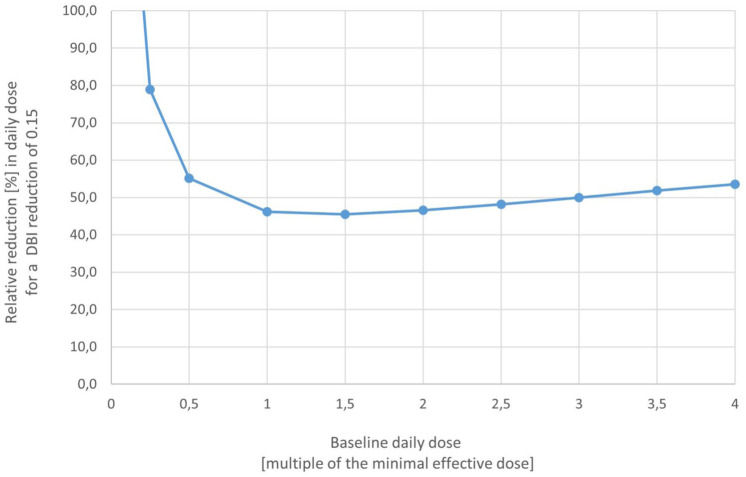
Required percentage reduction in daily dose for a DBI change of 0.15 by baseline daily dose. DBI, Drug Burden Index.

The DBI quantifies the total exposure to PSA-PIM at the patient level and is calculated from the sum of the DBIs of the individual substances, which in turn are calculated as follows

$$DBI=\sum \frac{D}{D\;+\;\;\delta }$$

where *D* is the daily dose, and δ is the minimum effective daily dose for each medication, which is used as an estimate of the *DR*_*50*_ (daily dose required to achieve 50% of maximal contributory effect at steady state).^33^ Figure 2 shows that for patients taking the minimum effective daily dose or a higher dose at baseline, a reduction of the DBI by 0.15 points is consistent with a dose reduction of one PSA-PIM by approximately 50%. In patients taking lower daily doses, larger relative dose reductions are required. Given the dose-dependency of sedative and anticholinergic effects, a DBI reduction of ⩾0.15 points is considered to be clinically meaningful.^32,34^ The specific PSA-PIMs and minimal effective doses considered to calculate the primary endpoint have been prespecified and are available from the authors upon request.

##### Secondary outcomes

Secondary endpoints are designed to provide a more detailed understanding of intervention effects on deprescribing of PSA-PIMs, other (i.e., non-PSA) PIMs, clinical and patient-reported outcomes, and costs.

Table 6 shows secondary outcomes measuring PSA-PIM deprescribing and use. SO1 to SO5 measure whether the effect on the primary endpoint is sustained at 12 months follow-up (SO1) and whether it differs between different groups of PSA-PIMs, that is, (SO2) chronic pain psychotropics (opioids and gabapentinoids), (SO3) mental health psychotropics (antidepressants and antipsychotics at doses exceeding those used to treat insomnia, see Table S1), (SO4) sedative-hypnotics (benzodiazepines, z-drugs, antihistamines, certain antidepressants and antipsychotics at doses lower than those used to treat mental health conditions), (SO5) anticholinergic urological antispasmodics. The impact of the intervention on the overall number of PSA-PIMs prescribed is measured by SO6, whereas SO7 measures its impact on reductions in baseline PIM use and SO8 its impact on new PSA-PIM prescribing.

Table 7 shows secondary outcomes SO9 to SO17 related to the use of PIMs other than those specifically targeted by PARTNER, as well as PPOs. The hypothesis to be tested here is that the medication review conducted by community pharmacists in the PARTNER intervention arm leads to optimized detection of MRPs (through the provision of patient information by the GP, the educational material provided in the web-based portal, and use of the PIM/PPO checklist) as well as corrective action to resolve them (through intensified collaboration between community pharmacists and GPs facilitated by the interprofessional workshop and planned feedback of medication review findings to GPs) compared to the eTAU control arm (where no particular tools or guidance are provided to support the medication check-up and feedback to GPs remains at the discretion of community pharmacists). The specific measures for SO9 to SO17 have been prespecified and are available from the authors upon request.

Table 8 shows patient-reported outcomes (SO18 to SO20) measuring any desired impact of the intervention on cognition (measured by the Verbal Fluency Test (VFT)^35,36^) and quality of life (EuroQol 5-Dimension 5-Level (EQ-5D-5L) questionnaire^37^), as well as any undesired impact of the intervention on insomnia (measured by the Regensburg Insomnia Scale (RIS)^38^).

Table 9 shows clinical outcomes (SO21 to SO23) measuring any desired impact of the intervention on falls with or without subsequent hospital admission. This is measured by FIMA (Questionnaire for Health-Related Resource Use in an Elderly Population)^39,40^ and a self-reported patient diary. The ADRs are measured by a ‘Patient-Reported Outcome Measure, Inquiry into Side Effects’ (PROMISE) ADR questionnaire^41^ modified for our study.

All patient questionnaires used as secondary outcome measures in our cRCT (see Supplemental Material) have been validated, either through established psychometric procedures (FIMA,^39,40^ EQ-5D-5L,^37,42^ RIS,^38^ VFT^35,36^) or via content validation involving both patients and clinical experts (ADR questionnaire^41,43^).

#### Sample size

Sample size calculation is based on the primary endpoint, the proportion of patients with a relevant reduction in DBI of at least 0.15 points at T_2_ (6 months after T_0_) compared to T_0_. Based on a Dutch intervention study^44^ and a slight positive effect expected in the control arm, we estimate the proportion of patients with a relevant reduction in DBI in the control arm to be 10%. Based on the successful Canadian EMPOWER study,^14^ whose main intervention component (patient empowerment by pharmacists) we adopt in the PARTNER intervention, we expect the proportion of patients with a relevant reduction in DBI to be 25% in the intervention arm. This corresponds to an absolute risk difference of 15% and an odds ratio (OR) of 3. Furthermore, we estimate the intra-cluster correlation coefficient (ICC) to be 0.05 and the average cluster size to be *n* = 6 patients. To be able to show a difference in responder rates of 10% versus 25% with a power of 80% at a significance level *α* = 0.05 with an ICC = 0.05, 22 clusters per group with a total of *n* = 264 patients are required. Under the conservative assumption of a maximum loss-to-follow-up of 25%, 352 patients (8 patients per cluster) will be recruited. Sample size was calculated using R software (version 4.1.1; R Foundation for Statistical Computing, Vienna, Austria).

#### Randomization

**Figure 3.**
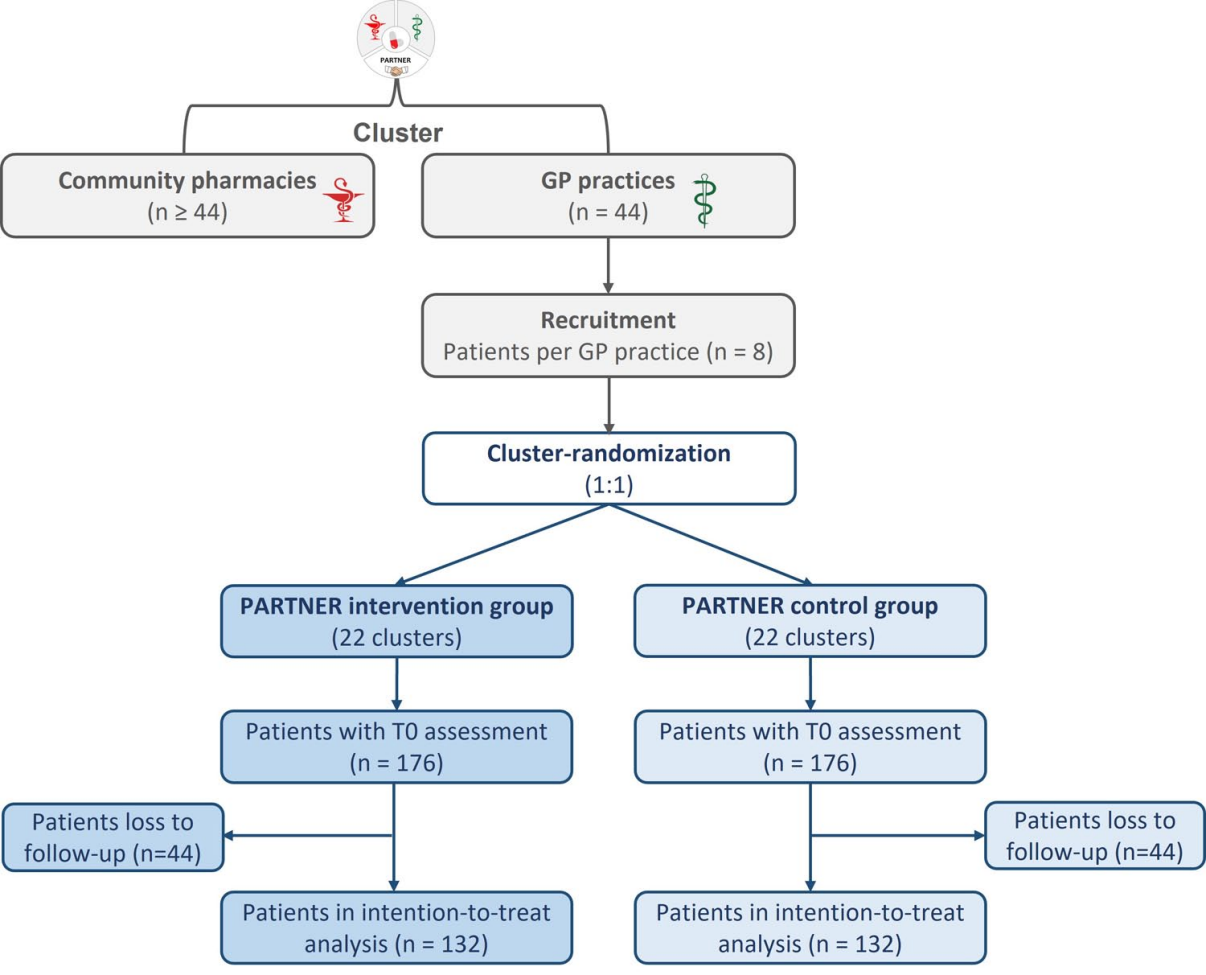
Flow chart according to CONSORT. CONSORT, Consolidated Standards of Reporting Trials; GP, general practitioner.

A cluster randomization at the level of the cooperation units, consisting of a GP practice and one or more community pharmacies, will be performed. Thus, all patients within a GP practice belong to the same group (either intervention or control group). The randomization is stratified by study center (Munich, Bielefeld, Witten), and the intervention and control groups are randomized in a 1:1 ratio. The random allocation sequence will be generated by the Center for Clinical Studies Regensburg by block randomization using SAS (version 9.4; SAS Institute Inc., Cary, NC, USA) and the procedure *proc plan*. Randomization lists will be stored at the Center for Clinical Studies and are concealed from the study centers until the end of the study. Allocation of a cluster to a treatment group will be directly after completion of patient recruitment within the cluster (see Figure 3).

#### Statistical analysis

A generalized linear mixed model (GLMM) is used for the analysis of the primary binary endpoint (reduction of ⩾0.15 points on the DBI at 6 months follow-up). In this model, the patients are nested under the cooperation units (clusters) with a random intercept for each cluster, whereby the number of degrees of freedom is determined using the between-within method.^45^ The GLMM is adjusted for study center and the known prognostic factors age, sex, and type and number of PSA-PIM at time T_0_. ORs with associated 95% confidence intervals are reported as effect sizes. In additional sensitivity analyses, the GLMM is adjusted for other covariates potentially associated with the primary endpoint.^46^ Subgroup analyses are planned to identify any differential effects of the intervention on different PSA-PIM drug groups. The study population for the primary analysis is based on the intention-to-treat principle, with a per-protocol analysis performed as a secondary analysis. Secondary endpoints will be presented descriptively and analyzed exploratively using the appropriate methods. All planned statistical analyses (including handling of missing data) will be prespecified in a Statistical Analysis Plan before the end of the study, which will be accessible at the Center for Clinical Studies Regensburg.

#### Study organization

The steering committee comprises staff of the LMU University Hospital and lead investigators of all participating study centers, and is responsible for the design, integrity, and progress of the trial. The steering committee is also tasked with implementing any potential protocol modifications.

The risks of the study to patients are considered to be low, as all patients will continue to receive regular care from their GP, regardless of their participation in the study. Nevertheless, a Data Monitoring Committee (DMC) that is independent of the study team and the study sponsor and has no conflicts of interest has been appointed to carry out safety-related tasks. If adverse symptoms occur during a deprescribing attempt, the GP may, at their own discretion, discontinue the attempt at any time and return to the original dose. If any unexpected serious events are identified during data collection by study personnel, these will be documented and forwarded to the DMC for review. The persons responsible for data collection at the respective study center will be trained to recognize these events and report them to the medical study personnel at the respective study center, who will then contact the GP if necessary. The DMC consists of two physicians with specific expertise in clinical pharmacology.

The Center for Clinical Studies Regensburg is responsible for data management and data quality assurance within the study. Details on data management and data protection are outlined in the data protection concept, which was submitted to the ethics committee alongside the ethics application and approved by the data protection officers at all study sites collecting data.

#### Dissemination

Within 1 year after completion of data collection, we will report results in ClinicalTrials.gov, including a flow chart of study recruitment and dropout, demographic and baseline characteristics of participants, primary and secondary outcomes, statistical test results, and adverse event information, if applicable. To make results available, we will provide the following: (1) Final report of the study results to the funder; (2) publication of study results in peer-reviewed journals; (3) presentations at national and international conferences; (4) plain language summary sent to patient participants.

### Health economic evaluation

#### Aims and objectives

The aim of the health economic evaluation is to provide evidence to inform decisions about the potential wider implementation of the intervention. While the ultimate decision is a political one, health economic analysis offers robust scientific insights to support informed policymaking.

Standard criteria for health economic evaluations will be assessed. To justify implementation into routine care, the intervention should meet at least one of the following conditions:

- The PARTNER intervention reduces healthcare costs for statutory health insurance (SHI) without negatively impacting patients’ quality of life.
- The PARTNER intervention improves patients’ quality of life without increasing healthcare costs.
- The PARTNER intervention enhances quality of life, with associated healthcare cost increases deemed acceptable within cost-effectiveness thresholds.

#### Study design

Primary outcomes from biometric analysis, such as reductions in the DBI and changes in the incidence of falls, will be compared between the control group and intervention group. Furthermore, for all study participants, health-associated quality of life is derived from the EQ-5D-5L questionnaire. Based on that, we compute the EQ-5D index as a summary statistic that is used to compare life quality developments between the IG and the CG. These outcomes will then be analyzed alongside corresponding changes in healthcare costs for both groups. We approximate those based on the study participants self-reported healthcare utilization and up-to-date respective unit costs for Germany. A detailed description of the methodological approach is given below.

#### Data collection and outcome measures

Table 10 outlines health economic outcomes (HEO1 to HEO5), including the cost and benefit endpoints considered in the analysis.

*Health-related utility*: Health-related utility is measured across five dimensions:

- *DBI*: Calculated based on patient interviews.
- *EQ-5D index*: Measures health-related quality of life using data from the EQ-5D-5L questionnaire, deriving a summary statistics from time-tradeoff analysis.^37,42^
- *EQ-VAS*: Measures health-related quality of life using data from the EQ-5D-5L questionnaire based on a visual analog scale.
- *Incidence of falls*: Captured through patient diaries.
- Cognition index: Assessed via a VFT.

*Cost analysis*: Intervention costs are categorized into *direct* and *indirect* costs:

- *Direct costs* are estimated based on documented expenses for informational materials and the time invested by GPs, nursing staff, and pharmacists related to the intervention. Time units are multiplied by corresponding cost units, derived from current reports and published unit costs.
- *Indirect costs* are assessed by collecting data on healthcare utilization over 3-month periods for both study groups. This includes physician visits, outpatient or inpatient hospital care, and physical or occupational therapies. Data is gathered using a validated questionnaire for Germany.^39,40^ Healthcare utilization is then multiplied by cost units, as done for direct cost estimation.

#### Statistical analysis

The health economic evaluation follows a three-step procedure previously recommended for health economic analyses in Germany.^47^ Cost analysis employs a *difference-in-differences* (DiD) approach to compare trends in individual healthcare cost estimates between the intervention group and the control group, incorporating both direct and indirect costs.

In the first step, utility analysis is conducted using the DiD method to compare changes across the five utility dimensions at the individual level. Utility differentials are calculated between the four survey periods and subsequently compared between the IG and CG using hypothesis testing. For instance, for a patient *i*, the first differences in some utility index between periods 0 and 1 are

$$\Delta u_{i,1}:=u_{i,1}-u_{i,0},$$

With *μ*_i,0_ being the observation for the respective utility index at *T*_0_ for patient *i*. We check the null hypothesis that the utility differentials are not associated with whether the patients belong to the IG or the CG, that is, for

$$\Delta u_{i,t}=\delta I+\beta \prime x_{i},$$

we check

$$H_{0}:\delta =0.$$

Here, *I* is a binary variable that takes the value 1 for patients of the IG and 0 for those in the CG. ***x***_i_ $x_{i}$ is a vector of sociodemographic predictors. Those are included to avoid a potential bias in the test results because of heterogeneities between the two study groups. If the null hypothesis of interest can be rejected, the intervention is associated with different utilities than the standard treatment. The Greeks are parameters estimated based on the data via ordinary least squares.

If the intervention does not lead to a deterioration in health utility, cost analysis will estimate cost differentials between the intervention and control groups using an approach similar to that applied for the utility dimensions. If the initial steps of the analysis do not demonstrate clear dominance of one care alternative over the other (i.e., neither intervention nor control prevails), cost-effectiveness analysis will be conducted, computing an incremental cost-effectiveness ratio (ICER). Analysis is conducted from the perspective of the SHI community, with the time horizon covering at least the study observation period. To address statistical uncertainty, stochastic sensitivity analyses will be performed using Monte Carlo simulations. These simulations generate empirical distributions of the ICER by simulating utility and cost indices 10,000 times. This approach provides an ICER distribution that can be used to estimate the probability of the intervention meeting prespecified cost-effectiveness thresholds, similar to methods described in Gillespie et al.^48^

ICERs will be calculated for all five utility dimensions to provide a comprehensive basis for healthcare decision-making. However, given the recognized importance of the EQ-5D index in health economic evaluations, recommendations for implementing the intervention into standard care will be primarily based on its cost-effectiveness analysis. The Monte Carlo simulations generate empirical distributions for both the utility (denominator) and cost (numerator) components of the ICERs based on 10,000 simulations of the overall cost and utilities change associated with the intervention through the observed period *T*_0_–*T*_4_. The nuisance parameters used for this are estimated by the earlier-mentioned regression models. For instance, the ICER in the trajectory *s* is:

$${\mathrm{ICER}}_{s}=\frac{\Delta u_{0-4,s,\mathrm{IG}}-\Delta u_{0-4,s,\mathrm{CG}}}{\Delta c_{0-4,s,\mathrm{IG}}-\Delta c_{0-4,s,\mathrm{CG}}},$$

with

-
$\Delta \mu_{0–4,s,\mathrm{IG}}$ : average utility differential between *T_0_* and *T_4_* for intervention group in trajectory *s*,
-
$\Delta \mu_{0–4,s,\mathrm{CG}}$ : average utility differential between *T_0_* and *T_4_* for control group in trajectory *s*,
-
$Dc_{0–4,s,\mathrm{IG}}$ : average cost differential between *T_0_* and *T_4_* for intervention group in trajectory *s*,
-
$Dc_{0–4,s,\mathrm{CG}}$ : average cost differential between *T_0_* and *T_4_* for control group in trajectory *s*.

This approach generates a non-parametric distribution for the ICER, which can be compared directly to predefined cost-effectiveness thresholds and provide a recommendation, if the implementation of the intervention is given some willingness to pay.

### Process evaluation

#### Aims and objectives

The overarching goal of the process evaluation is to gain a comprehensive understanding of why and how the intervention succeeded or failed. To achieve this, both quantitative and qualitative data will be systematically collected from participants and extracted from the main trial’s study documentation.

#### Frameworks

The process evaluation follows the framework for process evaluations of cluster-randomized trials developed by Grant et al.,^49^ which outlines key steps of intervention implementation: participant recruitment, intervention delivery, and participant response at both the cluster and individual patient levels. Barriers and facilitators to sustaining the intervention over time, as well as any unanticipated effects, will be explored. To systematically identify and structure the barriers, facilitators, and mechanisms influencing intervention delivery, response, and maintenance, we will apply the updated Consolidated Framework for Implementation Research (CFIR),^50^ which encompasses five domains: innovation, inner setting, outer setting, characteristics of individuals, and the implementation process.

#### Research questions

Table 11 outlines the distinct research questions addressed by the process evaluation, investigated using both quantitative and qualitative methods.

**Table 11.** Overview of research questions to be examined by the process evaluation of the PARTNER cluster-randomized trial by intervention level and process step.

Process step	Research question(s)	Themes and parameters to be examined qualitatively or quantitatively (data collection from “main study” documents or bespoke “process evaluation”)
Cluster level		
Recruitment of clusters	How were clusters recruited?	Qualitative (inductive and deductive): Procedure and deviations, preselection, reasons for (non-) participation, support from study staff (process evaluation)
		Quantitative: Recruitment rate: proportion (%) of participating/invited doctors and pharmacists (main study)
	. . . how high is the willingness to participate?	Quantitative: Recruitment rate: proportion (%) of participating/invited GPs and pharmacists (main study)
	. . . what reasons play a role in participation?	Qualitative (inductive): Reasons for participation/non-participation (process evaluation)
	. . . who is taking part in the study?	Quantitative: Comparison of characteristics of participants/non-participants (main study)
Intervention delivery and response	. . . how were the intervention components implemented by the study staff?	Qualitative (inductive): Experience of the study staff from the training courses, level of knowledge, and previous experience of the doctors and pharmacists, necessary deviations from the specified procedure (process evaluation)
		Quantitative: type, number, and “severity” of deviations (main study)
	. . . what support was required to enable the service providers to implement the intervention components?	Qualitative: Type and effort of support provided by study staff (process evaluation)
		Quantitative: Number of contacts between study personnel and service providers (main study)
	. . . how are intervention components implemented?	Quantitative: Descriptive (number/type of empowerment brochures per patient; main study)
		Quantitative: Number/spectrum of identified ABPs in medication analyses (main study)
		Quantitative: Number of doctor/pharmacist contacts per patient (main study)
		Quantitative: Proportion (%) of patients with ⩾1 deprescribing attempt (main study)
		Quantitative: Number of (PSA-PIM-related) contacts with doctor/pharmacist (main study)
Maintenance at cluster level	. . . how do GPs/pharmacists assess the benefits and importance of intervention components? (How) do they influence behavior?	Qualitative (inductive): Justification of importance and usefulness/suggestions for improvement; Information on usefulness in semi-structured interviews (process evaluation)
	. . . what barriers do GPs/pharmacists identify for deprescribing, collaboration and intervention roll-out?	Information on implementation barriers in semi-structured interviews (process evaluation)
Patient level		
Recruitment of patients	. . . how high is the willingness to participate?	Recruitment rate: Proportion (%) of participating/invited GPs and pharmacists (main study)
	. . . who is taking part in the study?	Quantitative: Comparison of characteristics of participants/non-participants (main study)
Intervention delivery and response	. . . how are intervention components adopted?	Quantitative: Proportion (%) of patients who state that they were aware of the empowerment brochure on first contact with the pharmacist (main study)
Maintenance at patient level	. . . how do patients assess the benefits and importance of intervention components?	Qualitative (inductive): Justification of the importance and usefulness/Suggestions for improvement (process evaluation)
	. . . what barriers do patients identify to deprescribing and rolling out the intervention?	Qualitative (deductive and inductive): Type and significance of named implementation barriers/Proposed solutions (process evaluation)

GP, general practitioner; PSA-PIM, psychotropic, sedating, and anticholinergic potentially inappropriate medication.

##### Cluster level

At the cluster level, characterizing participating clusters is essential to assess the external validity and generalizability of findings to other settings. Sociodemographic data will be collected, and experiences with GP-pharmacist collaboration will be evaluated across all clusters. Recruitment strategies will be documented by research staff, and the reasons and motivations for participation will be assessed for each cluster. To gain a deeper understanding of intervention delivery, response, and sustainability, we will examine how intervention components were implemented across clusters and how study tasks were distributed between GPs and pharmacists.

##### Patient level

Similarly, understanding the characteristics of patients included in the study is vital for assessing the generalizability of results. This includes details such as the specific PSA-PIMs patients are using at baseline, the conditions for which these medications were prescribed, and the cognitive status of participants. We will also investigate the extent to which pharmacists and GPs engaged with patients and how patients interacted with the intervention. For instance, we will evaluate the usage and comprehension of empowerment brochures and their potential influence on intervention effectiveness.

##### Cross-cutting assessment

At both the cluster and patient levels, we will examine participants’ perceptions of the intervention’s overall utility, including the relative importance of its components and the extent to which each component achieved its intended effects. Barriers and facilitators influencing the implementation of intervention components will also be identified at both levels, providing insights into factors that impacted success or challenges in delivery.

#### Study design

The process evaluation adopts a mixed-methods approach, combining written questionnaires (administered to all participants at six time points) with in-depth semi-structured interviews conducted with purposively sampled participants. For further details, please refer to Figure S1 in the Supplemental Material.

#### Sampling and data collection

##### Cluster level

All GPs and community pharmacists will be surveyed at baseline and following the individual intervention components using written questionnaires integrated into their study folders. A final written survey is sent to the GPs and pharmacists at the end of the study, allowing them to complete and submit the survey online via a provided link. The final questionnaire will be developed based on online scoping interviews with pre-selected clusters, chosen by the study teams based on their implementation performance during the trial. Participation in the final survey requires prior consent, obtained as part of the broader study consent process.

To gain deeper insights into recruitment and sampling strategies, semi-structured interviews will also be conducted with research staff involved in recruitment and organizational processes across the three study sites in Germany (Munich, Bielefeld, and Witten).

##### Patient level

All patients will be surveyed by research staff via telephone at baseline and at the end of the study. Based on the results, selected patients (up to 10%) from both the intervention and control groups will be invited to participate in additional telephone interviews. These interviews will provide deeper insights into participants’ reasons for joining the study and their behavior throughout the trial.

All qualitative data collection will occur prior to the end of the cRCT, and researchers will remain unaware of the intervention effects at the time of data collection, minimizing potential biases during the semi-structured interviews.

#### Statistical analysis

Data from the semi-structured interviews with study teams and patients will undergo qualitative analysis. Interviews with the study teams will be transcribed and analyzed using thematic analysis, clustering responses to specific questions. Patient interviews will be transcribed verbatim and analyzed using Kuckartz’s qualitative analysis method.^51^

Questionnaire data will be analyzed quantitatively and descriptively. Free-text responses relating to barriers or facilitators in implementing, delivering, or sustaining the intervention or its components will be coded, clustered, and mapped to the constructs of the CFIR. The process evaluation will compare results between the intervention and control groups and include longitudinal assessments using within-cluster and within-subject comparisons. Findings related to the implementation, delivery, and sustainability of intervention components will be correlated with overall intervention effectiveness.

Through both qualitative and quantitative analysis, we aim to identify diverse perspectives and potential correlations across findings. This approach will enable the systematic identification and evaluation of factors that facilitate or hinder the successful implementation of the intervention.

## Discussion

### Summary of key elements of the study

The PARTNER cluster-randomized trial evaluates a complex intervention aimed at deprescribing psychotropic, sedative, and anticholinergic potentially inappropriate medications (PSA-PIMs) in older adults within German primary care. Building on the Canadian EMPOWER intervention, which successfully increased hypnotic-sedative deprescribing, the intervention comprises comprehensive educational materials and tools for healthcare providers, patient empowerment initiatives (including carefully developed, lay-friendly written material reinforced during pharmacy consultations), and a strong focus on GP-pharmacist collaboration with distinct and supplementary roles within the deprescribing process. A health economic and process evaluation alongside the main trial is conducted to inform broader implementation of the PARTNER intervention if shown to be effective.

### Limitations

The PARTNER trial possesses several strengths related to its design, addressing key barriers to deprescribing psychotropic, sedative, and anticholinergic medications (PSA-PIMs) in older adults. However, potential limitations should also be acknowledged for each component of the intervention, with mitigations noted where applicable.

#### Collaboration between GPs and pharmacists

A key strength of the PARTNER intervention is its emphasis on fostering interprofessional collaboration between GPs and pharmacists. By integrating the medication review process into a collaborative framework, the intervention ensures that both professional groups deliver consistent and aligned messages to patients, which enhances trust and coordination. This approach addresses the common issue of fragmented working relationships that can lead to professional tensions. However, the effectiveness of this collaboration may be influenced by local communication practices and existing relationships between GPs and pharmacists. While the structured workshops aim to mitigate these tensions, varying dynamics may affect implementation. Despite this, the PARTNER model could offer valuable insights for healthcare systems with similar interprofessional challenges.

#### Educational materials and tools for healthcare providers

Another strength is the provision of comprehensive educational resources for GPs and pharmacists, which include practical tools for designing and operationalizing the tapering process. This additional support helps to alleviate professional uncertainty, enabling healthcare providers to guide patients confidently through the tapering of medications that require careful dose reduction. A limitation to consider is, however, the potential variability in engagement with these educational resources. Nevertheless, the reinforcement of educational messages and availability of tools during workshops are intended to increase the chance that these materials are used to help build both knowledge and confidence in deprescribing.

#### Patient empowerment initiatives

These lay-friendly empowerment brochures are intended to educate patients about the risks and benefits of their medications and prepare them for meaningful discussions with their GPs. However, frail and cognitively impaired patients may not fully understand or engage with these materials. The educational messages and arguments are therefore to be reinforced by pharmacists during consultations, which is intended to save GPs time during appointments by ensuring patients are informed and ready to engage. Nevertheless, some patients may still resist deprescribing, particularly if they perceive significant benefits from their medications.

#### Strategies to reduce risk of bias

A significant strength of the study design is the blinding of the control group with respect to the specific drugs targeted by the intervention. This blinding is crucial for avoiding contamination bias, as it prevents control clusters and patients from altering their behavior due to awareness of the study focus. Measures have been implemented to maintain this blinding throughout the recruitment of clusters and patients, ensuring they agreed to participate without knowledge of the intervention’s focus on PSA-PIMs. This means that intervention clusters are only informed of the intervention focus post-randomization, emphasizing the importance of the educational components and deprescribing tools in securing buy-in from GPs and pharmacists. The risk that clusters may accidentally treat intervention and control group patients (e.g., because they consult with different GP practices or community pharmacies) is minimized by maintaining sufficient geographical distance between recruited clusters. In addition, structural characteristics of the German pharmacy system further reduce the likelihood of cross-group information exchange. In Germany, pharmacists rarely work as “locums” in geographically disparate pharmacies. In addition, to minimize the risk of contamination bias between the intervention and control groups, particular care is taken to ensure that the details of the intervention are not disclosed during scientific meetings or professional conferences that may include GPs or pharmacists from the geographical areas where the study is conducted.

#### Selection of measurement instruments

For the assessment of some of the secondary patient-reported outcomes, more comprehensive questionnaires than those we selected are available. In particular, alternatives to the VFT—such as the Montreal Cognitive Assessment^52^ or the Mini-Mental State Examination^53^— may provide broader coverage or greater sensitivity to age-related cognitive changes. However, these instruments are considerably more time-consuming. Given the already high questionnaire burden on patient participants, our choice of measurement tools for secondary endpoints was strongly guided by the aim of keeping the assessments as concise and feasible as possible.

#### Process evaluation

The inclusion of a process evaluation alongside the main trial is another strength. The process evaluation aims to monitor the implementation of the intervention, assess factors influencing success, and identify opportunities for optimization. Although the process evaluation does not assess long-term sustainability, it provides valuable insights into the short-term implementation and effectiveness of the intervention, enabling adjustments for broader application.

### Implications for healthcare practice and research

The PARTNER trial’s focus on comprehensive educational resources and practical tools for healthcare providers highlights the need for ongoing professional development in managing complex medication regimens. The emergence of deprescribing networks and guidelines in several countries is an important development in this regard. However, practical tools equipping professionals and patients to plan and implement deprescribing processes may additionally be required to increase their confidence and efficacy in deprescribing.

In addition to better equipping healthcare professionals and patients deprescribing, the PARTNER trial evaluates the potential benefits of closer interprofessional collaboration between GPs and pharmacists in deprescribing efforts. Given the widening gap between healthcare supply and demand, it will be essential to fully utilize the knowledge and skills of all healthcare professionals and to develop models to integrate their efforts, which is particularly relevant in healthcare systems where primary healthcare providers operate in silos. The PARTNER trial tests one such collaborative model within the context of this trial, where collaboration is facilitated by the research team. If shown to be effective, further research should develop and test strategies (such as financial incentives for both GPs and pharmacists) to motivate such collaboration without the need for external facilitation.

## Conclusion

The PARTNER trial represents a significant advancement in the field of deprescribing by addressing key barriers that are specific to PSA-PIMs. Compared to previous trials, such as EMPOWER^14^ and D-PRESCRIBE,^54^ the PARTNER intervention provides more comprehensive educational resources to healthcare professionals and emphasizes the importance of collaborative communication between GPs and pharmacists. These innovations are designed to increase healthcare provider engagement and ensure that consistent messages are delivered to patients, which is critical for overcoming patient resistance to deprescribing. By focusing on shared decision-making, careful dose tapering, and professional collaboration, PARTNER addresses the unique challenges of deprescribing medications that patients may perceive as essential to their well-being. To the extent that the intervention’s educational and collaborative components are successfully implemented, the PARTNER trial has the potential to significantly enhance the impact of deprescribing efforts, not only within the German healthcare system but also in other settings where similar challenges exist.

## Supplemental Material

sj-docx-2-taw-10.1177_20420986251400042 – Supplemental material for General practitioner–pharmacist collaboration to enhance deprescribing of psychotropics, sedatives, and anticholinergics among older polypharmacy patients in primary care: study protocol of a cluster-randomized controlled trial (PARTNER) Supplemental material, sj-docx-2-taw-10.1177_20420986251400042 for General practitioner–pharmacist collaboration to enhance deprescribing of psychotropics, sedatives, and anticholinergics among older polypharmacy patients in primary care: study protocol of a cluster-randomized controlled trial (PARTNER) by Annette Haerdtlein, Kerstin Bernartz, Sophie Peter, Laura K. Lepenies, Svetlana Puzhko, Yvonne Eberhardt, Michaela Maas, Stephanie Picker-Huchzermeyer, Vita Brisnik, Diana Falomir, Jochen Gensichen, Tim Steimle, Gunnar Huppertz, Michael Koller, Florian Zeman, Patrizio Vanella, Hanna M. Seidling, Achim Mortsiefer, Christiane Muth and Tobias Dreischulte in Therapeutic Advances in Drug Safety

sj-jpg-3-taw-10.1177_20420986251400042 – Supplemental material for General practitioner–pharmacist collaboration to enhance deprescribing of psychotropics, sedatives, and anticholinergics among older polypharmacy patients in primary care: study protocol of a cluster-randomized controlled trial (PARTNER) Supplemental material, sj-jpg-3-taw-10.1177_20420986251400042 for General practitioner–pharmacist collaboration to enhance deprescribing of psychotropics, sedatives, and anticholinergics among older polypharmacy patients in primary care: study protocol of a cluster-randomized controlled trial (PARTNER) by Annette Haerdtlein, Kerstin Bernartz, Sophie Peter, Laura K. Lepenies, Svetlana Puzhko, Yvonne Eberhardt, Michaela Maas, Stephanie Picker-Huchzermeyer, Vita Brisnik, Diana Falomir, Jochen Gensichen, Tim Steimle, Gunnar Huppertz, Michael Koller, Florian Zeman, Patrizio Vanella, Hanna M. Seidling, Achim Mortsiefer, Christiane Muth and Tobias Dreischulte in Therapeutic Advances in Drug Safety

sj-pdf-1-taw-10.1177_20420986251400042 – Supplemental material for General practitioner–pharmacist collaboration to enhance deprescribing of psychotropics, sedatives, and anticholinergics among older polypharmacy patients in primary care: study protocol of a cluster-randomized controlled trial (PARTNER) Supplemental material, sj-pdf-1-taw-10.1177_20420986251400042 for General practitioner–pharmacist collaboration to enhance deprescribing of psychotropics, sedatives, and anticholinergics among older polypharmacy patients in primary care: study protocol of a cluster-randomized controlled trial (PARTNER) by Annette Haerdtlein, Kerstin Bernartz, Sophie Peter, Laura K. Lepenies, Svetlana Puzhko, Yvonne Eberhardt, Michaela Maas, Stephanie Picker-Huchzermeyer, Vita Brisnik, Diana Falomir, Jochen Gensichen, Tim Steimle, Gunnar Huppertz, Michael Koller, Florian Zeman, Patrizio Vanella, Hanna M. Seidling, Achim Mortsiefer, Christiane Muth and Tobias Dreischulte in Therapeutic Advances in Drug Safety

## Declarations

**Ethics approval and consent to participate:** The PARTNER study (protocol version 1.2) and associated study materials have been approved by the Ethics Committee of the Medical Faculty of LMU Munich (date: 16 November 2022, reference: 22-0713) and by the local ethics committees of the Medical Association of Westphalia-Lippe and the Westphalian Wilhelms-University Münster (date: 07 December 2022, reference: 2022-774-b-S), the University of Witten/Herdecke (date: 15 December 2022, reference: 255/2022), and the Medical Faculty of the University of Heidelberg (date: 21 December 2022, reference: S-747/2022). All participating general practitioners, pharmacists, and patients provided written informed consent to participate in the PARTNER trial.

**Consent for publication:** Not applicable.

**Availability of data and materials:** Not applicable.
