# Supplementary material for: General practitioner–pharmacist collaboration to enhance deprescribing of psychotropics, sedatives, and anticholinergics among older polypharmacy patients in primary care: study protocol of a cluster-randomized controlled trial (PARTNER)
Source: Ther Adv Drug Saf. 2026 Jan 8;17:20420986251400042. doi: 10.1177/20420986251400042 (PMC12783581; doi:10.1177/20420986251400042)
Supplement: sj-docx-2-taw-10.1177_20420986251400042 – Supplemental material for General practitioner–pharmacist collaboration to enhance deprescribing of psychotropics, sedatives, and anticholinergics among older polypharmacy patients in primary care: study protocol of a cluster-randomized controlled trial (PAR [file sj-docx-2-taw-10.1177_20420986251400042.docx]

**Table S1: List of PSA-PIMs sorted by drug class and indication**

|  |  | Drugs used as sedative-hypnotics | Psychotropics  used to treat mental health conditions | Psychotropics used to treat chronic pain | Anticholinergics used to treat urological conditions |
| --- | --- | --- | --- | --- | --- |
| Antidepressants | ATC code |  |  |  |  |
| **Non-selective monoamine reuptake inhibitors** | **N06AA** |  |  |  |  |
| Imipramine | N06AA02 |  | **✓** |  |  |
| Clomipramine | N06AA04 |  | **✓** |  |  |
| Opipramol | N06AA05 | **✓** |  |  |  |
| Trimipramine | N06AA06 | **✓** (≤ 50 mg) | **✓** (> 50 mg) |  |  |
| Amitriptyline | N06AA09 | **✓** (≤ 50 mg) | **✓** (> 50 mg) |  |  |
| Nortriptyline | N06AA10 |  | **✓** |  |  |
| Doxepin | N06AA12 | **✓** (≤ 50 mg) | **✓** (> 50 mg) |  |  |
| Maprotiline | N06AA21 |  | **✓** |  |  |
| Amitriptylinoxide | N06AA25 | **✓** (≤ 50 mg) | **✓** (> 50 mg) |  |  |
| **Selective serotonin reuptake inhibitors** | **N06AB** |  |  |  |  |
| Fluoxetine | N06AB03 |  | **✓** |  |  |
| Citalopram | N06AB04 |  | **✓** |  |  |
| Paroxetine | N06AB05 |  | **✓** |  |  |
| Sertraline | N06AB06 |  | **✓** |  |  |
| Fluvoxamine | N06AB08 |  | **✓** |  |  |
| Escitalopram | N06AB10 |  | **✓** |  |  |
| **Monoamine oxidase inhibitors, non-selective** | **N06AF** |  |  |  |  |
| Tranylcypromine | N06AF04 |  | **✓** |  |  |
| **Other antidepressants** | **N06AX** |  |  |  |  |
| Mianserin | N06AX03 |  | **✓** |  |  |
| Trazodone | N06AX05 | **✓** (≤ 100 mg/d) | **✓** (> 100 mg/d) |  |  |
| Mirtazapine | N06AX11 | **✓** (≤ 15 mg) | **✓** (> 15 mg) |  |  |
| Bupropion | N06AX12 |  | **✓** |  |  |
| Tianeptine | N06AX14 |  | **✓** |  |  |
| Venlafaxine | N06AX16 |  | **✓** |  |  |
| Milnacipran | N06AX17 |  | **✓** |  |  |
| Duloxetine | N06AX21 |  | **✓** |  |  |
| Agomelatine | N06AX22 |  | **✓** |  |  |
| Antipsychotics |  |  |  |  |  |
| **Phenothiazines with aliphatic side-chain** | **N05AA** |  |  |  |  |
| Levomepromazine | N05AA02 | **✓** (≤ 25 mg/d) | **✓** (> 25 mg/d) |  |  |
| **Phenothiazines with piperazine structure** | **N05AB** |  |  |  |  |
| Fluphenazine | N05AB02 |  |  |  |  |
| Perphenazine | N05AB03 |  |  |  |  |
| Perazine | N05AB10 |  |  |  |  |
| **Phenothiazines with piperidine structure** | **N05AC** |  |  |  |  |
| Thioridazine | N05AC02 |  |  |  |  |
| **Butyrophenone derivatives** | **N05AD** |  |  |  |  |
| Haloperidol | N05AD01 |  | **✓** |  |  |
| Melperone | N05AD03 | **✓** (≤ 75 mg/d) | **✓** (> 75 mg/d) |  |  |
| Pipamperone | N05AD05 | **✓** (≤ 40 mg/d) | **✓** (> 40 mg/d) |  |  |
| Benperidol | N05AD07 |  | **✓** |  |  |
| **Indole derivatives** | **N05AE** |  |  |  |  |
| Ziprasidone | N05AE04 |  | **✓** |  |  |
| **Thioxanthene derivatives** | **N05AF** |  |  |  |  |
| Flupentixol | N05AF01 |  | **✓** |  |  |
| Chlorprothixen | N05AF03 | **✓** |  |  |  |
| Zuclopenthixol | N05AF05 |  | **✓** |  |  |
| **Diphenylbutylpiperidine derivatives** | **N05AG** |  |  |  |  |
| Fluspirilene | N05AG01 |  | **✓** |  |  |
| **Diazepines, oxazepines, thiazepines and oxepines** | **N05AH** |  |  |  |  |
| Clozapine | N05AH02 |  | **✓** |  |  |
| Olanzapine | N05AH03 |  | **✓** |  |  |
| Quetiapine | N05AH04 | **✓** (≤ 100 mg/d) | **✓** (> 100 mg/d) |  |  |
| **Benzamides** | **N05AL** |  |  |  |  |
| Sulpiride | N05AL01 |  | **✓** |  |  |
| Tiapride | N05AL03 |  | **✓** |  |  |
| Amisulpride | N05AL05 |  | **✓** |  |  |
| **Other antipsychotics** | **N05AX** |  |  |  |  |
| Prothipendyl | N05AX07 | **✓** |  |  |  |
| Risperidon | N05AX08 |  | **✓** |  |  |
| Aripiprazol | N05AX12 |  | **✓** |  |  |
| Paliperidon | N05AX13 |  | **✓** |  |  |
| Cariprazin | N05AX15 |  | **✓** |  |  |
| **Other hypnotics and sedatives** | **N05CM** |  |  |  |  |
| Clomethiazole | N05CM02 | **✓** |  |  |  |
| Promethazine | N05CM22 | **✓** |  |  |  |
| Benzodiazepines |  |  |  |  |  |
| **Benzodiazepine derivatives** | **N05BA** |  |  |  |  |
| Diazepam | N05BA01 | **✓** |  |  |  |
| Chlordiazepoxide | N05BA02 | **✓** |  |  |  |
| Medazepam | N05BA03 | **✓** |  |  |  |
| Oxazepam | N05BA04 | **✓** |  |  |  |
| Potassium clorazepate | N05BA05 | **✓** |  |  |  |
| Lorazepam | N05BA06 | **✓** |  |  |  |
| Bromazepam | N05BA08 | **✓** |  |  |  |
| Clobazam | N05BA09 | **✓** |  |  |  |
| Alprazolam | N05BA12 | **✓** |  |  |  |
| **Benzodiazepine derivatives** | **N05CD** |  |  |  |  |
| Flurazepam | N05CD01 | **✓** |  |  |  |
| Nitrazepam | N05CD02 | **✓** |  |  |  |
| Flunitrazepam | N05CD03 | **✓** |  |  |  |
| Triazolam | N05CD05 | **✓** |  |  |  |
| Lormetazepam | N05CD06 | **✓** |  |  |  |
| Temazepam | N05CD07 | **✓** |  |  |  |
| Midazolam | N05CD08 | **✓** |  |  |  |
| Brotizolam | N05CD09 | **✓** |  |  |  |
| **Benzodiazepine related drugs** | **N05CF** |  |  |  |  |
| Zopiclone | N05CF01 | **✓** |  |  |  |
| Zolpidem | N05CF02 | **✓** |  |  |  |
| **Benzodiazepine derivatives** | **N03AE** |  |  |  |  |
| Clonazepam | N03AE01 | **✓** |  |  |  |
| Gabapentinoids |  |  |  |  |  |
| Gabapentin | N02BF01 |  |  | **✓** |  |
| Pregabalin | N02BF02 |  |  | **✓** |  |
| Opioids |  |  |  |  |  |
| **Natural opium alkaloids** | **N02AA** |  |  |  |  |
| Oxycodone | N02AA05 |  |  | **✓** |  |
| Hydromorphone | N02AA03 |  |  | **✓** |  |
| Morphine | N02AA01 |  |  | **✓** |  |
| Dihydrocodeine | N02AA08 |  |  | **✓** |  |
| Oxycodone and naloxone | N02AA55 |  |  | **✓** |  |
| Codeine | N02AA59 |  |  | **✓** |  |
| **Phenylpiperidine derivatives** | **N02AB** |  |  |  |  |
| Pethidine | N02AB02 |  |  | **✓** |  |
| Fentanyl | N02AB03 |  |  | **✓** |  |
| **Oripavine derivatives** | **N02AE** |  |  |  |  |
| Buprenorphine | N02AE01 |  |  | **✓** |  |
| **Other opioids** | **N02AX** |  |  |  |  |
| Tramadol | N02AX02 |  |  | **✓** |  |
| Tapentadol | N02AX06 |  |  | **✓** |  |
| Tilidine and naloxone | N02AX51 |  |  | **✓** |  |
| Anticholinergic antispasmodics |  |  |  |  |  |
| **Drugs for urinary frequency and incontinence** | **G04BD** |  |  |  |  |
| Flavoxate | G04BD02 |  |  |  | **✓** |
| Oxybutynin | G04BD04 |  |  |  | **✓** |
| Propiverine | G04BD06 |  |  |  | **✓** |
| Tolterodine | G04BD07 |  |  |  | **✓** |
| Solifenacin | G04BD08 |  |  |  | **✓** |
| Trospium | G04BD09 |  |  |  | **✓** |
| Darifenacin | G04BD10 |  |  |  | **✓** |
| Fesoterodine | G04BD11 |  |  |  | **✓** |
| Desfesoterodine | G04BD13 |  |  |  | **✓** |

*Abbreviations:* PSA-PIM = psychotropic/sedative/anticholinergic potentially inappropriate medication; ATC = Anatomical Therapeutic Chemical (Classification System)
