# Supplementary figures and images for: General practitioner–pharmacist collaboration to enhance deprescribing of psychotropics, sedatives, and anticholinergics among older polypharmacy patients in primary care: study protocol of a cluster-randomized controlled trial (PARTNER)
Source: Ther Adv Drug Saf. 2026 Jan 8;17:20420986251400042. doi: 10.1177/20420986251400042 (PMC12783581; doi:10.1177/20420986251400042)

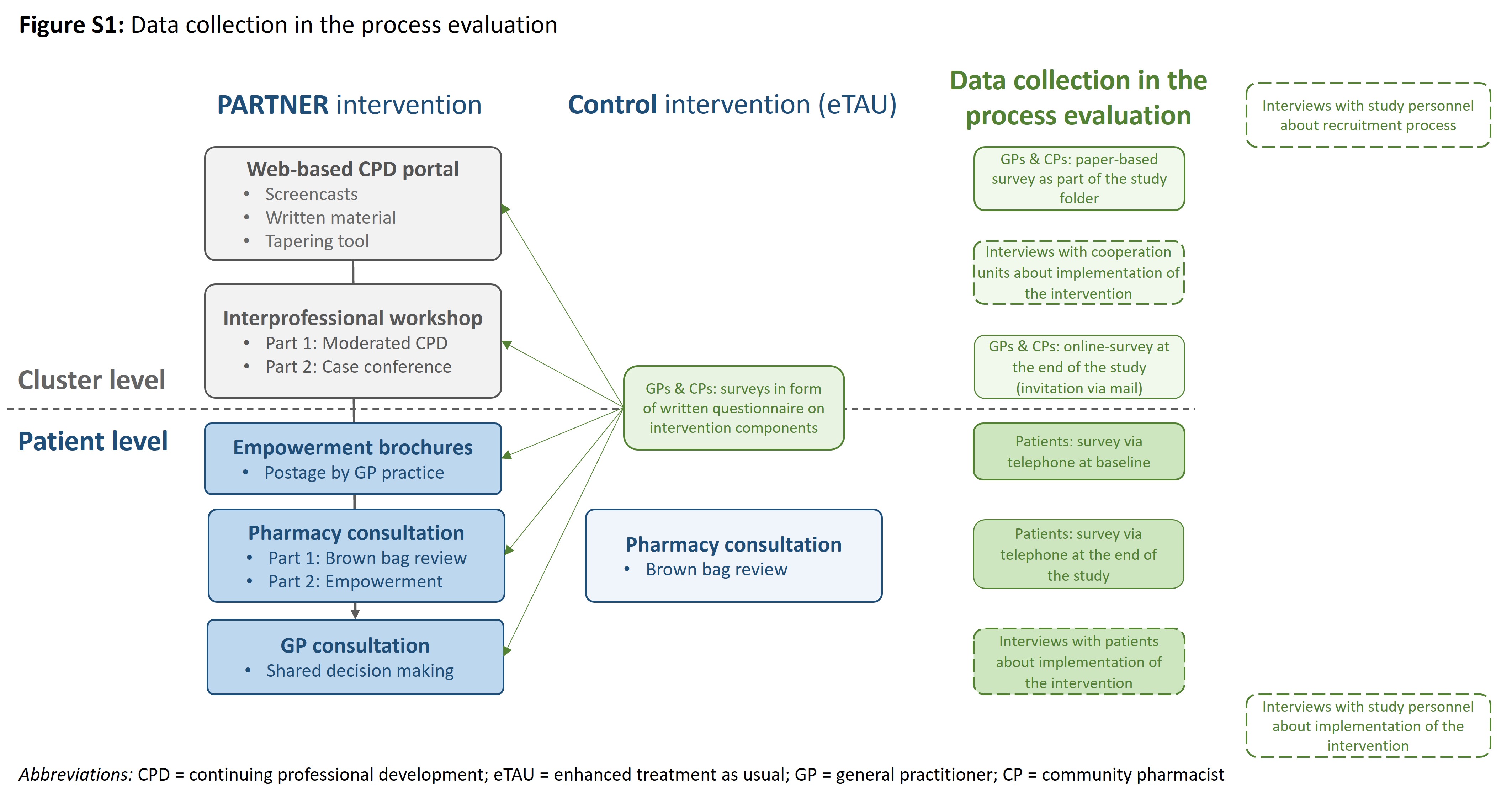

Supplement: sj-jpg-3-taw-10.1177_20420986251400042 – Supplemental material for General practitioner–pharmacist collaboration to enhance deprescribing of psychotropics, sedatives, and anticholinergics among older polypharmacy patients in primary care: study protocol of a cluster-randomized controlled trial (PART [file sj-jpg-3-taw-10.1177_20420986251400042.jpg]
