# Supplementary material for: General practitioner–pharmacist collaboration to enhance deprescribing of psychotropics, sedatives, and anticholinergics among older polypharmacy patients in primary care: study protocol of a cluster-randomized controlled trial (PARTNER)
Source: Ther Adv Drug Saf. 2026 Jan 8;17:20420986251400042. doi: 10.1177/20420986251400042 (PMC12783581; doi:10.1177/20420986251400042)
Supplement: sj-pdf-1-taw-10.1177_20420986251400042 – Supplemental material for General practitioner–pharmacist collaboration to enhance deprescribing of psychotropics, sedatives, and anticholinergics among older polypharmacy patients in primary care: study protocol of a cluster-randomized controlled trial (PART [file sj-pdf-1-taw-10.1177_20420986251400042.pdf]

Patient questionnaires

Please note: The following questionnaires are the English translation of the original German versions that were actually used in the study.

Patient-centred Deprescribing of Psychotropic, Sedative and Anticholinergic Medication in Elderly Patients with Polypharmacy (PARTNER)

Documentation of medication by the study team

Medication - Data collection: T0, T2, T4

Medication

Patient-ID				/				/			
Date of completion	_ _ . _ _ ._ _ _ _ (tt.mm.jjjj)										

Current Medication

Active ingredient	Brand name	Strength	Dosage form	Dosage schedule				Unit	Instructions	Indication
				morning	noon	evening	at night			

Medication

Patient-ID				/				/			
Date of completion	_ _ . _ _ . _ _ _ _ (tt.mm.jjjj)										

I hereby confirm that the information provided is correct and complete.

Date, Signature: ____

Patient-centred Deprescribing of Psychotropic, Sedative and Anticholinergic Medication in Elderly Patients with Polypharmacy (PARTNER)

FIMA questionnaire

(General questions)

FIMA: Health-related resource use in an elderly population: general questions - Data collection: T0

Patient-ID			
Date of completion	_ _ . _ _ . _ _ _ _ (tt.mm.jjjj)		

General questions
What is your gender?<br>☐ Male<br>☐ Female<br>☐ Diverse
In which year were you born?<br><br>_______________________________
What is your marital status?<br>☐ married<br>☐ single<br>☐ divorced<br>☐ widowed
What is your **highest level** of school education?<br>☐ Lower secondary school (Hauptschule/Volksschule)<br>☐ Intermediate secondary school (Mittlere Reife/Realschule)<br>☐ Upper secondary school (Abitur/Fachabitur/University entrance qualification)<br>☐ No school-leaving certificate<br>☐ Other qualification: ____
What is your **highest** vocational qualification?<br>☐ Vocational training (apprenticeship)<br>☐ Technical school/Master craftsman school<br>☐ Engineering school/polytechnic<br>☐ University/university of applied sciences<br>☐ No vocational qualification<br>☐ Other qualification:____
Where do you currently live?<br>☐ Private household<br>☐ Senior apartment (assisted living, senior residence)<br>☐ Residential home for older adults<br>☐ Nursing home
What type of health insurance do you have?<br>☐ Statutory health insurance<br>☐ Government aid plus private insurance (civil servant status)<br>☐ Private health insurance (full coverage, not supplementary insurance)<br>☐ No health insurance
With which health insurance company are you insured?<br><br>____
Is German your native language?<br><br>☐ Yes ☐ No, my native language is: ____<br><br>If **no**: How many years have you been living in Germany?<br>____

I hereby confirm that the information provided is correct and complete.

Date, Signature: ____

Patient-centred Deprescribing of Psychotropic, Sedative and Anticholinergic Medication in Elderly Patients with Polypharmacy (PARTNER)

FIMA questionnaire

(Further questions)

FIMA: Health-related resource use in an elderly population - Data collection: T0, T1, T2, T3, T4

FIMA

Patient-ID			
Date of completion	_ _ . _ _ . _ _ _ _ (tt.mm.jjjj)		

Do you receive benefits from the statutory long-term care insurance?
☐ No
☐ Yes
If yes, what level of care do you have, or how high is your monthly care allowance?<br><br>Care level: ________<br>Or<br>Care allowance: ____

Do you own one or more of the following assistive devices?			
Assistive device	No	Yes, I have been using this for a longer time	Yes, used within the last 12 weeks
Walker (rollator)	☐	☐	☐
Wheelchair	☐	☐	☐
Stair lift	☐	☐	☐
Bathtub lift	☐	☐	☐
Glasses / visual aid	☐	☐	☐
Hearing aid / hearing device	☐	☐	☐
Dental prothesis	☐	☐	☐
Oxygen device	☐	☐	☐
Sleep apnea treatment	☐	☐	☐
Compression stockings	☐	☐	☐
Incontinence pads (regularly required)	☐	☐	☐
Other:___________________________	☐	☐	☐

Have you used any of the following services in the past month?			
**Therapeutic service**	**No**	**Yes**	**How often?**
Physiotherapy (including physical therapy, massages, heat or cold treatments, electrotherapy, or therapeutic baths)	☐	☐	
Alternative practitioner (Heilpraktiker)	☐	☐	
Osteopath	☐	☐	

In the past month, have you used a home care nursing service due to your health condition?
☐ No
☐ Yes
If yes, on how many days per week or per month did the home care service come to your home?<br><br>_________days per week<br><br>_________days per month.
On those days, how long was the home care secive at your home on average?<br><br>On average _____________hours, _____________ minutes

Patient-ID			
Date of completion	_ _ . _ _ . _ _ _ _ (tt.mm.jjjj)		

**In the past month, have you had a stay in a day-care (partially inpatient) care facility?**
☐ No
☐ Yes
If yes, how many days in total did you spend in the day-care facility?<br><br>______days

**In the past month, have you had a stay in an inpatient short-term care facility?**
☐ No
☐ Yes
If yes, how many days in total did you spend in the short-term care facility?<br><br>______days

**In the past month, have you had an outpatient or inpatient rehabilitation (rehabilitation stay or follow-up rehabilitation) in a rehabilitation facility?**
☐ No
☐ Yes
If yes, was your rehabilitation outpatient or inpatient?<br>☐ Outpatient<br>☐ Inpatient<br><br>The rehabilitation treatment lasted: _______ days

**In the past month, have you had a stay in a hospital?**
☐ No
☐ Yes
If yes, was one of the following reasons the cause for the hospital admission?<br>☐ Injury due to a fall<br>☐ Seizure<br>☐ Psychological problems<br>☐ Confusion<br>☐ Other ____
**In the past three months, have you additionally had a stay in a hospital?**
☐ No
☐ Yes
If yes, was one of the following reasons the cause for the hospital admission?<br>☐ Injury due to a fall<br>☐ Seizure<br>☐ Psychological problems<br>☐ Confusion<br>☐ Other ____

FIMA

Patient-ID			
Date of completion	_ _ . _ _ . _ _ _ _ (tt.mm.jjjj)		

Only from T1 (not yet at baseline survey/T0!):

**Did you complete the diary on your own?**
☐ Yes
☐ No
If no, who helped you?<br>☐ Relative<br>☐ Friend/acquaintance<br>☐ Caregiver
**If yes, how often did you complete the diary?**
☐ Daily<br>☐ Almost daily<br>☐ Once per week<br>☐ Twice per week<br>☐ Once per month<br>☐ Less than once per month
**How much time did you need to complete the diary?**
**Diary:** ___________minutes in the past month

**Please estimate how difficult it was for you to complete the diary:**
☐ Very easy<br>☐ Easy<br>☐ Difficult<br>☐ Very difficult<br>☐ Impossible without help

I hereby confirm that the information provided is correct and complete.

Date, Signature: ____

Patient-centred Deprescribing of Psychotropic, Sedative and Anticholinergic Medication in Elderly Patients with Polypharmacy (PARTNER)

EQ-5D-5L

EQ-5D-5L: Health-related quality of life -

Data collection: T0, T1, T2, T3, T4

The English version of the EQ-5D-5L questionnaire can be viewed here:
EuroQol Group

Gesundheitsfragebogen

Deutsche Version für Deutschland

(German version for Germany)

Sample

Germany (German) © 2009 EuroQol Group EQ-5D™ is a trade mark of the EuroQol Group

Bitte kreuzen Sie unter jeder Überschrift DAS Kästchen an, das Ihre Gesundheit HEUTE am besten beschreibt.

BEWEGLICHKEIT / MOBILITÄT

Ich habe keine Probleme herumzugehen ❑

Ich habe leichte Probleme herumzugehen ❑

Ich habe mäßige Probleme herumzugehen ❑

Ich habe große Probleme herumzugehen ❑

Ich bin nicht in der Lage herumzugehen ❑

FÜR SICH SELBST SORGEN

Ich habe keine Probleme, mich selbst zu waschen oder anzuziehen ❑

Ich habe leichte Probleme, mich selbst zu waschen oder anzuziehen ❑

Ich habe mäßige Probleme, mich selbst zu waschen oder anzuziehen ❑

Ich habe große Probleme, mich selbst zu waschen oder anzuziehen ❑

Ich bin nicht in der Lage, mich selbst zu waschen oder anzuziehen ❑

ALLTÄGLICHE TÄTIGKEITEN *(z.B. Arbeit, Studium, Hausarbeit, Familien- oder Freizeitaktivitäten)*

Ich habe keine Probleme, meinen alltäglichen Tätigkeiten nachzugehen ❑

Ich habe leichte Probleme, meinen alltäglichen Tätigkeiten nachzugehen ❑

Ich habe mäßige Probleme, meinen alltäglichen Tätigkeiten nachzugehen ❑

Ich habe große Probleme, meinen alltäglichen Tätigkeiten nachzugehen ❑

Ich bin nicht in der Lage, meinen alltäglichen Tätigkeiten nachzugehen ❑

SCHMERZEN / KÖRPERLICHE BESCHWERDEN

Ich habe keine Schmerzen oder Beschwerden ❑

Ich habe leichte Schmerzen oder Beschwerden ❑

Ich habe mäßige Schmerzen oder Beschwerden ❑

Ich habe starke Schmerzen oder Beschwerden ❑

Ich habe extreme Schmerzen oder Beschwerden ❑

ANGST / NIEDERGESCHLAGENHEIT

Ich bin nicht ängstlich oder deprimiert ❑

Ich bin ein wenig ängstlich oder deprimiert ❑

Ich bin mäßig ängstlich oder deprimiert ❑

Ich bin sehr ängstlich oder deprimiert ❑

Ich bin extrem ängstlich oder deprimiert ❑

Sample

Germany (German) © 2009 EuroQol Group EQ-5D™ is a trade mark of the EuroQol Group

- Wir wollen herausfinden, wie gut oder schlecht Ihre Gesundheit HEUTE ist.
- Diese Skala ist mit Zahlen von 0 bis 100 versehen.
- 100 ist die beste Gesundheit, die Sie sich vorstellen können. 0 (Null) ist die schlechteste Gesundheit, die Sie sich vorstellen können.
- Bitte kreuzen Sie den Punkt auf der Skala an, der Ihre Gesundheit HEUTE am besten beschreibt.
- Jetzt tragen Sie bitte die Zahl, die Sie auf der Skala angekreuzt haben, in das Kästchen unten ein.

IHRE GESUNDHEIT HEUTE = ☐

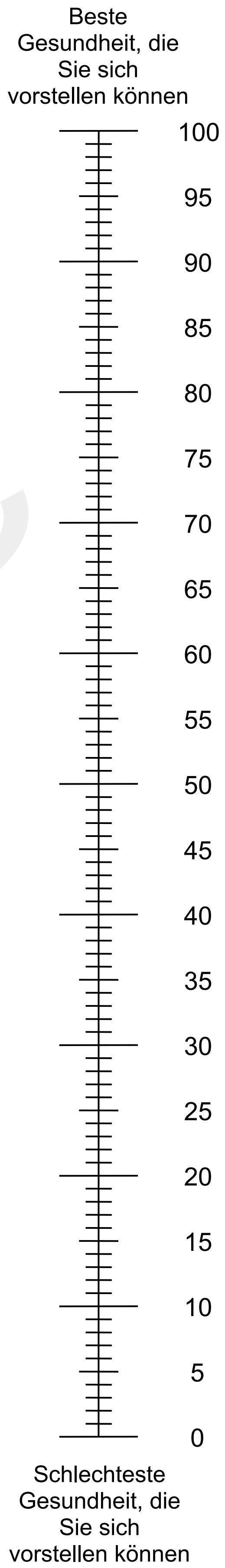

Sample

Germany (German) © 2009 EuroQol Group EQ-5D™ is a trade mark of the EuroQol Group

Patient-centred Deprescribing of Psychotropic, Sedative and Anticholinergic Medication in Elderly Patients with Polypharmacy (PARTNER)

Verbal Fluency Test

Verbal Fluency Test: Cognition -

Data collection: T0, T2, T4

Patient-ID			
Date of completion	_ _ . _ _ . _ _ _ _ (tt.mm.jjjj)		

Please list as many different animals as possible within one minute.
________________________(number per minute)

I hereby confirm that the information provided is correct and complete.

Date, Signature: ____

Patient-centred Deprescribing of Psychotropic, Sedative and Anticholinergic Medication in Elderly Patients with Polypharmacy (PARTNER)

Regensburg Insomnia Scale

Regensburg Insomnia Scale (RIS) -
Data collection: T0, T2, T4

Date:__________ Pat.-ID:___________________________

REGENSBURG INSOMNIA SCALE (RIS)

The following questions are intended to help your treating therapist assess your sleep difficulties. Please answer the questions by placing a checkmark next to the most appropriate answer. Only one checkmark per question or statement is allowed. The questions refer to the past four weeks.

0. My usual bedtime is:	from ....PM to ....AM on the next day	

1. How many minutes do you usually need to fall asleep?	**1-20 min.**	**20-40 min.**	**40-60 min.**	**60-90 min.**	**more than 90 min.**
	0	*1*	*2*	*3*	*4*
2. How many hours do you think you sleep on average per night?	**7+**	**5-6**	**4**	**2-3**	**0-1**
	0	*1*	*2*	*3*	*4*

	**always**	**mostly**	**sometimes**	**rarely**	**never**
3. I cannot sleep through the night.	*4*	*3*	*2*	*1*	*0*
4. I wake up too early.	*4*	*3*	*2*	*1*	*0*
5. I wake up at the slightest noise.	*4*	*3*	*2*	*1*	*0*
6. I feel as if I haven’t slept a wink all night.	*4*	*3*	*2*	*1*	*0*
7. I think a lot about my sleep.	*4*	*3*	*2*	*1*	*0*
8. I am afraid to go to bed because I worry I won’t be able to sleep.	*4*	*3*	*2*	*1*	*0*
9. I feel fully capable during the day.	*0*	*1*	*2*	*3*	*4*
10. I take sleeping medication to be able to fall asleep.	*4*	*3*	*2*	*1*	*0*

© 2013 T. Crönlein

Patient-centred Deprescribing of Psychotropic, Sedative and Anticholinergic Medication in Elderly Patients with Polypharmacy (PARTNER)

ADR questionnaire

Adverse Drug Reactions (ADRs) -

Data collection: T0, T2, T4

ADR questionnaire

	In the past four weeks, have you experienced any of the following symptoms?	0	1	If yes: Are these symptoms related to a chronic condition or medications you were already taking?	If yes: How often did you experience the following symptoms (in the last 4 weeks)?	0	1	2	3	If answer 1 or 2: How much do these symptoms bother you?	0	1	2	3	4
1	**Headaches**	No	Yes			On one day	On several days	Almost every day	Every day		Not at all	A little	Mode rately	Quite a lot	Very much
2	**Dizziness, tendency to fall, balance problems**	No	Yes			On one day	On several days	Almost every day	Every day		Not at all	A little	Mode rately	Quite a lot	Very much
3	**Fatigue, daytime sleepiness**	No	Yes			On one day	On several days	Almost every day	Every day		Not at all	A little	Mode rately	Quite a lot	Very much
4	**General weakness, reduced stamina, rapid exhaustion**	No	Yes			On one day	On several days	Almost every day	Every day		Not at all	A little	Mode rately	Quite a lot	Very much
5	**Mood swings**	No	Yes			On one day	On several days	Almost every day	Every day		Not at all	A little	Mode rately	Quite a lot	Very much
6	**Eye problems (burning, dry eyes, feeling of sand in the eyes), visual disturbances, blurred vision**	No	Yes			On one day	On several days	Almost every day	Every day		Not at all	A little	Mode rately	Quite a lot	Very much
7	**Dry mouth (dry mucous membranes, dry tongue, difficulty speaking or swallowing)**	No	Yes			On one day	On several days	Almost every day	Every day		Not at all	A little	Mode rately	Quite a lot	Very much
8	**Changes in appetite: increased or decreased appetite**	No	Yes			On one day	On several days	Almost every day	Every day		Not at all	A little	Mode rately	Quite a lot	Very much
9	**Weight gain**	No	Yes			Not applicable	Not applicable	Not applicable	Not applicable		Not at all	A little	Mode rately	Quite a lot	Very much
10	**Heart burn**	No	Yes			On one day	On several days	Almost every day	Every day		Not at all	A little	Mode rately	Quite a lot	Very much
11	**Abdominal pain**	No	Yes			On one day	On several days	Almost every day	Every day		Not at all	A little	Mode rately	Quite a lot	Very much
12	**Nausea or vomiting**	No	Yes			On one day	On several days	Almost every day	Every day		Not at all	A little	Mode rately	Quite a lot	Very much

13	**Constipation**	No	Yes		On one day	On several days	Almost every day	Every day		Not at all	A little	Moderately	Quite a lot	Very much
14	**Diarrhea**	No	Yes		On one day	On several days	Almost every day	Every day		Not at all	A little	Moderately	Quite a lot	Very much
15	**Bloating**	No	Yes		On one day	On several days	Almost every day	Every day		Not at all	A little	Moderately	Quite a lot	Very much
16	**Feeling of fullness**	No	Yes		On one day	On several days	Almost every day	Every day		Not at all	A little	Moderately	Quite a lot	Very much
17	**Difficulty urinating**	No	Yes		On one day	On several days	Almost every day	Every day		Not at all	A little	Moderately	Quite a lot	Very much
18	**Palpitations, skipped beats, or rapid heartbeat**	No	Yes		On one day	On several days	Almost every day	Every day		Not at all	A little	Moderately	Quite a lot	Very much
19	**Chills, shivering, or hand tremors**	No	Yes		On one day	On several days	Almost every day	Every day		Not at all	A little	Moderately	Quite a lot	Very much
20	**Increased sweating**	No	Yes		On one day	On several days	Almost every day	Every day		Not at all	A little	Moderately	Quite a lot	Very much
21	**Muscle or joint pain**	No	Yes		On one day	On several days	Almost every day	Every day		Not at all	A little	Moderately	Quite a lot	Very much
22	**Muscle weakness**	No	Yes		On one day	On several days	Almost every day	Every day		Not at all	A little	Moderately	Quite a lot	Very much
23	**Skin problems, itching, or dry skin**	No	Yes		On one day	On several days	Almost every day	Every day		Not at all	A little	Moderately	Quite a lot	Very much
24	**Increased tendency to bleed (e.g., nosebleeds, gum bleeding, large bruises [larger than a 2-Euro coin], prolonged bleeding from minor injuries such as shaving or kitchen work)**	No	Yes		On one day	On several days	Almost every day	Every day		Not at all	A little	Moderately	Quite a lot	Very much
25	**Other [+ free text field]**	No	Yes		On one day	On several days	Almost every day	Every day		Not at all	A little	Moderately	Quite a lot	Very much
25	**If two or more symptoms from questions 1–24 were reported:**								**How much are you overall affected by these problems?**	Not at all	A little	Moderately	Quite a lot	Very much
